# Stakeholders and Contextual Factors in the Implementation of Assistive Robotic Arms for Persons With Tetraplegia: Deductive Content Analysis of Focus Group Interviews

**DOI:** 10.2196/65759

**Published:** 2025-05-16

**Authors:** Vera Fosbrooke, Marco Riguzzi, Anja M Raab

**Affiliations:** 1 Bern University of Applied Sciences, School of Health Professions Bern Switzerland; 2 Center for Clinical Nursing Science, University Hospital Zurich University of Zurich, Institute of Implementation Science in Health Care Zurich Switzerland

**Keywords:** assistive technologies, robotic arm, implementation, barriers, facilitators, Consolidated Framework for Implementation Research

## Abstract

**Background:**

Tetraplegia imposes significant challenges on affected individuals, caregivers, and health care systems. Assistive technologies (ATs) such as assistive robotic arms have been shown to improve the quality of life of persons with tetraplegia, fostering independence in daily activities and reducing caregiver burden. Despite potential benefits, the integration of AT innovations into daily life remains difficult. Implementation science offers a systematic approach to bridge this know-do gap.

**Objective:**

This study aimed to (1) identify and involve relevant stakeholders; (2) identify relevant contextual factors (barriers and facilitators); and (3) suggest a general outlook for the implementation of AT, specifically an assistive robotic arm, into the everyday private lives of individuals with tetraplegia in Switzerland.

**Methods:**

A qualitative design was used, involving 3 semistructured online focus group interviews with 8 stakeholder groups, including persons with tetraplegia as well as those who could provide perspectives on engineering or technology, legal matters, nursing or care, therapy, social counseling, social insurance, and political considerations. The interviews were analyzed using the Focus Group Illustration Mapping tool, and the data were aligned with the domains of the Consolidated Framework for Implementation Research.

**Results:**

3 focus group interviews comprising 22 participants were conducted, and data were mapped onto 21 constructs across the Consolidated Framework for Implementation Research domains. Identified barriers were customization to users’ needs, safety concerns, and financing issues for the high AT costs. The collaboration with different stakeholders, including those who provided perspectives on political engagement, proved crucial. Identified facilitators included the enhancement of autonomy for persons with tetraplegia, improvement of quality of life, reduction of caregiver dependency, and addressing health care labor shortages. The implementation outlook involved the formation of an experienced team and the development of an implementation plan using hybrid type 1 and type 2 designs that incorporate both qualitative and quantitative implementation and innovation outcomes.

**Conclusions:**

Robotic arms offer promising benefits in terms of improved participation for users, while high costs and regulatory complexities as to who will assume these costs limit their implementation. These findings highlight the complexities involved in implementing AT innovations and the importance of addressing contextual factors. A specific framework for the implementation of AT is needed to ensure the successful integration in Switzerland and other countries with comparable social and health insurance systems.

## Introduction

### Background

Recent technological advancements have led to the proliferation of assistive technologies (ATs), including assistive robotic arms, improving the quality of life for individuals with spinal cord injury (SCI) [1]. SCI is a profound, life-altering condition that affects approximately 15.4 million people worldwide [2], with significant impacts on individuals, caregivers, and health care systems [3-6]. Tetraplegia, the partial or complete loss of motor and sensory function in all 4 extremities and the trunk, is a common manifestation of cervical SCI [7]. Upper extremity function ranks highest in importance among individuals with tetraplegia, often prioritized over functions such as bowel, bladder, sexual, or walking abilities [7]. Disabled upper extremities not only diminish independence and social participation but hinder employment prospects, leading to increased care costs [8,9]. Comparatively, individuals with SCI often experience lower quality of life levels than their able-bodied peers [10,11], and regardless of the specific nature of their impairments, they frequently depend on caregivers to meet their daily needs [12,13]. Paid caregiving services can impose a significant financial burden on either the individual or the funding agency [14], whereas unpaid caregiving, provided mainly by family members, results in significant psychological implications [11,15,16].

Persons with impaired upper extremities, whether due to tetraplegia or other causes, such as multiple sclerosis or cerebral palsy [17], heavily depend on ATs to enhance independence and participation in activities of daily living (ADLs) [18,19]. ATs augment individuals’ capabilities and broaden the range of activities they can engage in. Commonly used adaptations include cutlery, environmental control systems, writing orthoses [20], wheelchairs, prosthetics, and orthotics [21]. The application of AT not only aids the user but also indirectly benefits caregivers, notably family caregivers. Atoyebi et al [22] emphasize the potential of AT in reducing caregiver burden and alleviating stress. Specifically assistive robots, as defined by Kyrarini et al [17], serve as compensatory ATs and offer external assistance controlled by the user through an interface [23,24]. They facilitate a wide range of activities for persons with upper extremity disabilities, enhance care and promote independence across different areas [25,26]. One example is the Functional Robot with Dexterous Arm and User Friendly Interface for Disabled People (FRIEND) system, a wheelchair-mounted robotic manipulator designed to assist users with tetraplegia in tasks such as drinking and eating. The FRIEND IV, the latest iteration, integrates a 7-degree of freedom robotic arm and a 2-finger gripper and has been tested in a use-scenario, achieving a 95% success rate for cataloging books (of a performed 100 runs) with the end user’s intervention [27]. The Jaco 2 robotic arm (Kinova Inc), a widely used commercially available system, offers 6- or 7-degree of freedom configurations and can be equipped with 2- or 3-finger grippers. It has been applied in various contexts, such as adaptive feeding systems that integrate force sensors and cameras to detect and manipulate food for users. In an efficacy study with 31 participants, the Jaco robotic arm has demonstrated the potential to reduce caregiving time by approximately 41% [28]. Beyond feeding and drinking tasks, assistive robotic systems have also been implemented in other ADLs. The Baxter humanoid robot, for example, has been used to provide personalized dressing assistance, using force-minimizing methods to help users with upper-body impairments wear sleeveless jackets [29]. Other experimental systems have explored applications such as beard shaving, hair brushing, and bathing, although these tasks remain in early stages of development and require further research to ensure safety and usability [17]. However, most robotic systems are still in the research phase and require significant customization and personalization to effectively assist persons with impaired upper extremity in their daily life.

There have been efforts to promote high-performance AT as prototypes, such as international contests like the CYBATHLON hosted by the Swiss Federal Institute of Technology Zurich in Switzerland [30,31]. Nonetheless, the need [32,33], the transfer, and the adaptation of AT in everyday life remain a transnational challenge [1,34,35], and many robotic innovations fail to reach the market [36]. This discrepancy can be attributed to various factors, including a focus on technical feasibility over user needs, complexity, cost, and the need for technical assistance for use [36,37]. For effective market integration, factors such as reliability, cost-effectiveness, esthetics, functionality, and usability must be carefully considered, with user-centered design principles playing a relevant role [23,38,39].

### Objectives

The need of structured knowledge transfer, reimbursement strategies, and the assessment of the readiness of the AT market to successfully transition a prototype into a market-ready product [30] can be met with implementation sciences, offering valuable methods for the systematic uptake of evidence-based practices [40]. The implementation sciences acknowledge the relevant influence of economic, political, professional, and sociohistorical context factors on the adoption of evidence in practice [40]. The aim of this study is to investigate and reduce the research translation gap of implementing ATs into social and health insurance systems, with a specific focus on integrating a user-centered designed assistive robotic arm into the everyday private lives of individuals with tetraplegia in Switzerland [41]. The study’s objectives are (1) identifying and involving relevant stakeholders, (2) assessing contextual factors (barriers and facilitators), and (3) proposing an implementation strategy for the robotic arm.

## Methods

### Qualitative Design

A qualitative design was applied, conducting 3 online focus group interviews with representatives from 8 stakeholder groups relevant to the robotic arm’s implementation, aiming to identify barriers and facilitators. This study was performed from a constructivist or interpretivist point of view using a general deductive approach. This research was reported in accordance with the Standards for Reporting Qualitative Research by O’Brien et al [42].

### Conceptual Framework

The Consolidated Framework for Implementation Research (CFIR) serves as a widely used guide in implementation research, comprising 39 constructs synthesizing existing theories applicable across all implementation phases, allowing researchers to select the most relevant constructs for their specific study setting [43-45]. It aims to identify and explain factors influencing implementation outcomes, consisting of 5 major domains: innovation, outer setting, inner setting, individuals involved, and implementation process [45]. The innovation is related to the characteristics of the innovation being implemented into a particular setting. Outer and inner settings represent a dynamic interface, where the outer setting generally includes the political, social, and economic context, such as community and state factors, while the inner setting comprises the structural and cultural context. Individuals involved represents the individuals involved with the innovation and the implementation process. In this domain, cultural, organizational, and professional aspects as well as individual mindsets and norms are considered. The implementation process includes the development needed during implementation. According to Damschroder et al [46], researchers should choose the most relevant constructs from the 39 offered in the CFIR to suit their specific study context. These constructs can guide diagnostic evaluations of the implementation environment, monitor implementation progress, and interpret findings in the research studies or qualitative improvement initiatives.

### Context

The robotic arm at the center of the focus groups was a prototype developed by the Bern University of Applied Sciences in Switzerland. It competed in the Assistance Robot Race at the CYBATHLON 2024, mastering 10 different tasks of daily life, such as removing a parcel from the mailbox or navigating a touch screen. However, it has not yet been subjected to trials beyond this competition [47]. The robotic arm system consists of a Kinova Gen3 robotic arm with 7 joints, which can be mounted on an electric wheelchair. A 3D camera positioned near the gripper provides visual input for the current task. The vision system uses machine learning algorithms to identify objects, enabling the robot to approach and grasp them in a semiautomated manner, thereby saving time in completing the task. A graphical user interface displayed on a tablet near the user presents live and processed images from the camera. Users with some arm mobility can interact with the system by using the tablet’s touch screen to issue commands, while those without sufficient mobility rely on speech recognition powered by Whisper (OpenAI) [48]. The robotic arm system is designed to ensure simple operation and intelligent robotic behavior. The architecture of the system itself is independent of the robotic arm, allowing for flexibility in implementation. For instance, while the current configuration uses the Kinova Gen3 robotic arm, it could be replaced with an alternative model, such as the BATEO (ACCREA Engineering) arm, without compromising the system’s functionality. In addition to the existing literature on different robotic arm systems, a cross-sectional study focusing on the identification of needs for ATs for upper limbs in persons with tetraplegia was considered for the design of the robotic arm (N Hutmacher, unpublished data, March 2025). An overview of the robotic arm system can be found in Multimedia Appendix 1.

Representatives from 8 stakeholder groups (affected persons, as well as those who could provide perspectives on engineering or technology, legal matters, nursing or care, therapy, social counseling, social insurance, and political considerations) participated in the focus group interviews. The nursing or care stakeholder group included professional caregivers and family members who provided caregiving, the therapy group comprised occupational and physical therapists, and the social insurance group included representatives from various Swiss social insurance organizations (Swiss social insurers according to Federal Accident Insurance [UVG], Federal Military Insurance [MVG], and Federal Law on Invalidity Insurance [IVG]). Inclusion criteria required stakeholders to have specific professional roles or specialized knowledge, be aged >18 years, and have a good understanding of German.

### Sampling Strategy

Stakeholders were organized into focus groups, each consisting of 6 to 9 participants, with a minimum of 4 stakeholder groups represented in each focus group. This approach aimed to facilitate discussions and ensure diverse perspectives were represented among stakeholders in a heterogenous group composition [46,47].

Purposive sampling aimed to achieve maximum variation in specialty by using a snowball approach to contact official representatives from insurance institutions, government offices, medical clinics specializing in SCI, assistive product retailers, and personal contacts. Recruiting involved contact via email or phone by the main researcher [42].

The researchers found consistent discussion of similar barriers and facilitators after 3 focus group interviews, confirming data saturation.

### Data Collection Methods

To avoid increased time expenditure with long journeys, the 120-minute focus group interviews were conducted online via Microsoft Teams during January 2024 to February 2024.

Data collected for the general implementation outlook were informed by extensive literature research.

### Data Collection Instruments and Technologies

Data collection followed a semistructured interview guide (Multimedia Appendix 2) developed by the research team based on literature review. The interview guide was refined in 3 one-on-one online interviews with participants (including a representative of engineering or technology, legal perspective, and social insurers) who did not participate in the focus groups. The guide was supplemented by the discussion and answers from the participants in the one-on-one meetings. The final guide contained the areas first impressions, opportunities, barriers, dealbreakers, and outlook or solutions.

Ground rules, a schedule, and a photograph of the robotic arm as stimulus material (Multimedia Appendix 3) were introduced at the start of each focus group interview. Participants identified themselves by role and name, with sessions being video and audio recorded using the Microsoft Teams application. Moderation was handled by the main researcher, and note-taking was handled by a note-taker.

### Units of Study

A total of 3 focus group interviews were conducted with 6 to 9 representatives of the different stakeholder groups shown in Table 1 (focus group 1: 9/22, 41%; focus group 2: 6/22, 27%; and focus group 3: 7/22, 32%). A total of 7 stakeholder groups were represented in at least 1 focus group; no participant engaged in more than 1 group. Family caregivers from the nursing or care and political perspective groups did not participate in the study. Most study participants (13/22, 59%) were female. Participants appeared to be highly experienced in their professional roles; they had worked on average 15 (SD 9.23) years in the current professional function.

**Table 1:** Units of study in total and stratified per focus group (N=22).

	Participants, n (%)	Experience in years, mean (SD; minimum/maximum)	Participants in focus group 1, n (%)	Participants in focus group 2, n (%)	Participants in focus group 3, n (%)
Affected persons^a^	4 (18)	—^b^	2 (22)	1 (17)	1 (14)
Engineering or technology	5 (23)	14 (6.34; 6/20)	0 (0)	3 (50)	2 (29)
Legal matters	1 (4)	5 (—; 5/5)	1 (11)	0 (0)	0 (0)
Nursing or care	2 (9)	18 (19.80; 4/32)	1 (11)	1 (17)	0 (0)
Therapy^c^	5 (23)	14 (9.98; 5/25)	3 (33)	0 (0)	2 (29)
Social counseling	2 (9)	17 (4.24; 14/20)	2 (22)	0 (0)	0 (0)
Social insurance^d^	3 (14)	20 (11.72; 11/33)	0 (0)	1 (17)	2 (29)
Total	22 (100)	15 (9.23; 4/33)	9 (100)	6 (100)	7 (100)

^a^Disability years for the affected persons: mean=27; minimum/maximum: 5/46.

^b^Not applicable.

^c^Therapy included physiotherapists and occupational therapists.

^d^Swiss social insurers according to Federal Accident Insurance, Federal Military Insurance, and Federal Law on Invalidity Insurance.

### Data Processing

The Focus Group Illustration Mapping tool, as outlined by Pelz et al [49], facilitated a comprehensive summary of focus group interview content by combining survey methods with content evaluation. It used the semistructured interview guide as its framework, with key aspects first impressions, opportunities, barriers, dealbreakers, and outlook or solutions serving as central nodes. Relationships between concepts were represented using connecting arrows, with color codes distinguishing stakeholder groups. Visualizations were created using the web-based tool Miro [50], and PDF versions were sent to participants for consensual validation (Multimedia Appendices 4-6). The illustration maps were translated from German to English (Multimedia Appendices 7-9).

### Data Analysis

The CFIR constructs were selected after intensive examination of all 39 constructs according to the time of the study. A deductive approach was used to code data from the illustration maps, which were aligned with domains 1 to 4 of the CFIR framework.

For the implementation outlook, a similar deductive approach was used to code data extracted from literature research, focusing exclusively on the implementation process domain.

### Techniques to Enhance Trustworthiness

To ensure credibility of the findings, participant validation was used. A link to a Microsoft Forms survey allowed the validation of the illustration map by each participant anonymously, where either the option “I agree with the visual summary” or the option “I do not agree with the visual summary,” followed by an open text field for feedback, could be chosen (Multimedia Appendices 10 and 11). The response rate for the validation was 77%, with 17 participants completing the survey. The illustration maps were translated from German to English using 3 translation programs: DeepL (DeepL SE), LEO (LEO GmbH), and Dict.cc (dict.cc GmbH). Data triangulation was achieved through semistructured interviews and document analysis of online research, converging multiple data sources to validate identified themes and patterns [42].

### Ethical Considerations

Written informed consent was obtained from all participants outlining the investigation’s purpose, the use of data, and their publication (Multimedia Appendix 12). Given the design of the study and the nature of the data collected, formal ethics approval was not required. According to Risk Category A as defined in Article 7 of the Swiss Human Research Ordinance (HRO), studies involving noninvasive procedures and fully anonymized qualitative data, such as focus group interviews, are exempt from requiring ethics approval [51]. The research was conducted in accordance with ethical principles respecting the dignity, privacy, and autonomy of the participants.

## Results

**Figure 1:**
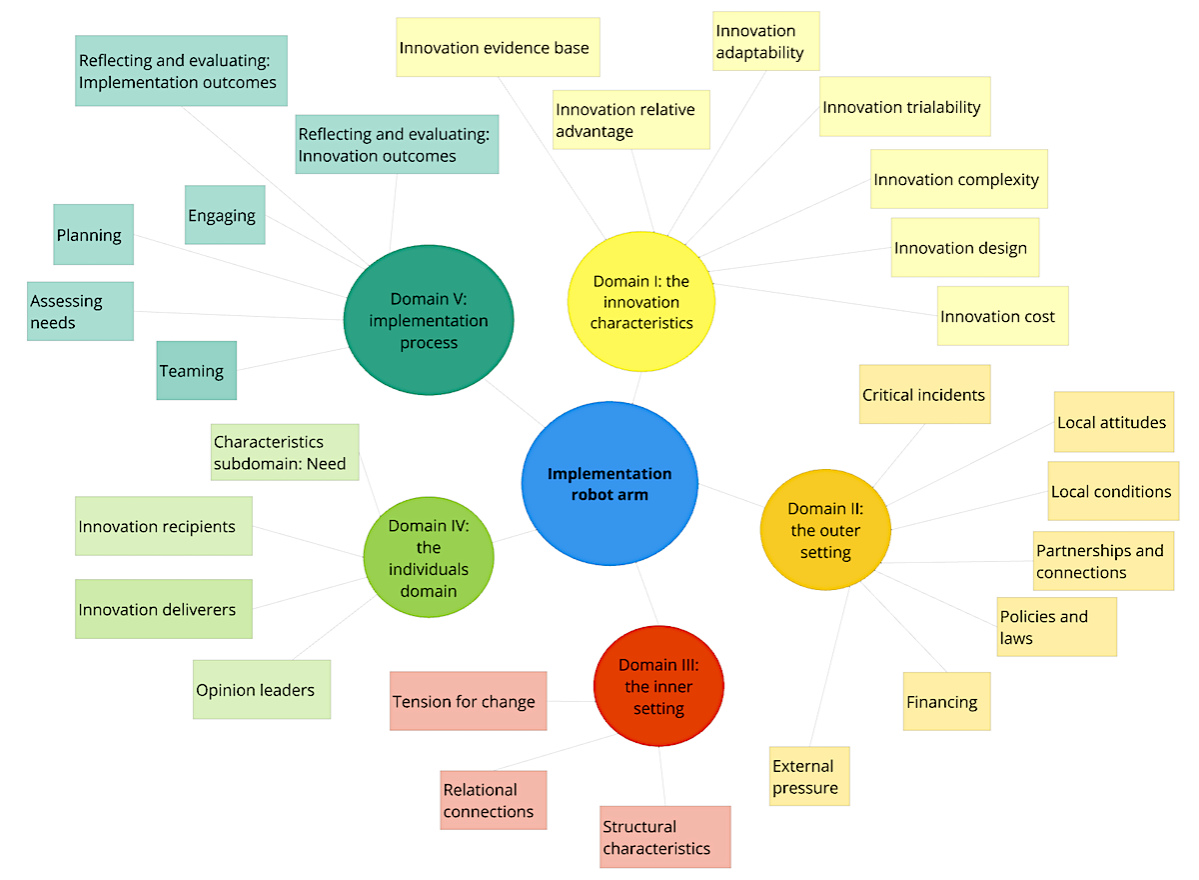
Overview of the selected Consolidated Framework for Implementation Research constructs.

The selected constructs of the 39 CFIR constructs for all 5 domains are displayed in Figure 1.

### Barriers and Facilitators to Implementation

#### Overview

Multimedia Appendix 13 summarizes the barriers and facilitators across 21 constructs within the CFIR domains innovation, outer setting, inner setting, and individuals domain. In the following, quotation marks are used to mark direct phrasing from the illustration maps.

#### Domain I: Innovation

##### Innovation Evidence Base

Facilitators that were mapped on the innovation evidence base could be supported with published literature. Overall, participants noted the robotic arm to enhance “self-care” and the facilitating aspect of increased quality of life through more autonomy [28,52]. In addition, it was reported that the robotic arm “saves on skilled labor” in the health care system, supporting its effectiveness [52]. Moreover, the robotic arm represented an “opportunity in the second labor market” [52].

##### Innovation Relative Advantage

According to participants, the robotic arm allowed an advantage over caregiver dependency, where simultaneously “autonomy is enabled.” Regarding this, one participant mentioned a potential negative aspect, namely, “there may be fewer human interactions.” The robotic arm’s inability to support critical activities such as transfers was noted, rendering users dependent on caregiver support. Participants mentioned reduced dependence on social insurance benefits, potentially enabling “work ability and integration.”

##### Innovation Adaptability

Identified barriers included the robotic arm’s inability to fit both electric and active wheelchairs and the challenge of customizing it to each user’s environment. Recognized opportunities to overcome these barriers were customization through the interface and the integration of a “combination with existing wearables,” noted as a facilitator by a technical stakeholder.

##### Innovation Trialability

Testing the robotic arm in everyday life and then having the opportunity to decide in favor of it was seen as necessary by the participants.

##### Innovation Complexity

The complex requirement for the robotic arm to replace the human arm function was seen as a challenge, where “technical limitations,” such as “recognizing material” and adjusting gripping force, were noted. However, one affected person emphasized functionality over speed of execution. Although the decision-making would always remain with the user when using the robotic arm, cognitive limitations were seen as a factor, highlighting the importance of the arm’s intuitiveness and ease of operation. The introduction of differing user profiles and operating modes, as well as the automatization of the robotic arm, were seen as facilitators for innovation complexity. This would allow for shared use of the robotic arm, for example, in “residential homes.” The interaction of the robotic arm with a dynamic human instead of a static object presented another barrier, with safety concerns, particularly regarding “spasticity” adding complexity to the innovation.

##### Innovation Design

he size and the one-sided weight of the robotic arm were perceived to affect the maneuverability of the electric wheelchair, potentially obstructing proximity to objects and hindering activities such as approaching a table. Concerns about the robotic arm’s “robustness” and “sensitivity” were raised due to potential damage from impacts or road conditions. The life expectancy of the robotic arm was considered a possible barrier, and features such as “material resistance to temperature, dust, and moisture” were mentioned as decisive. Environmental settings such as “car or taxi transport” or interactions with “young children” were also seen as potential hindrances to the innovation design. One participant noted that a “noisy environment” could affect the voice control of the robotic arm. The power regulation and dependency of the robotic arm were additional barriers identified. Some stakeholders found the robotic arm to be conspicuous, particularly when added to the already large wheelchair. However, affected persons prioritized practicality over aesthetics.

##### Innovation Cost

High costs of the robotic arm emerged as a significant barrier across all focus group interviews. The current estimated cost of the robotic arm system is around 50,000 Swiss francs (US $55,000), with additional expenses for customization, maintenance, repair, and indemnity insurance. “Savings on structural measures or technical aids” and the saving of resources as well as increasing work ability were noted as facilitators, potentially offsetting costs in the long term. Participants suggested expanding the target group to “other disorders with impaired upper extremity” or healthy individuals simplifying market entry and potentially lowering prices. However, this change in scope could affect assumptions about cost bearers.

#### Domain II: Outer Setting

##### Critical Incidents

Participants identified user involvement in both the development and financing processes, as well as public acceptance, as key factors that facilitate successful implementation. However, general dependency on technology and user helplessness in emergencies were seen as barriers, emphasizing the need for robust safety protocols and self-help capabilities. “WWarranty and availability of materials and spare parts” were critical barriers, and one stakeholder noted that “success depends on retention services, service intervals, duration of use, and guarantee pages.” “High costs” emerged as a significant barrier across all focus group interviews, emphasizing economic considerations and financial implications.

##### Local Attitudes

While some participants viewed technology, including robotics, as the future, concerns were raised about potential stigmatization. Furthermore, the need of political engagement to enhance social inclusion was stated by the participants, and it appeared relevant to manage expectations and clarify the robotic arm’s role as an assistive product.

##### Local Conditions

One facilitating factor noted was the possibility of using the robotic arm on public transportation in Switzerland, similar to how an electric wheelchair is used. Concerns were raised about potential damage to the robotic arm or injury to others during its use. In addition, it was mentioned that international travel with the robotic arm could be limited.

##### Partnerships and Connections

Collaborative opportunities, including partnerships with universities and “media partners,” and the involvement of therapists were seen as facilitating for the robotic arm’s implementation. One stakeholder representing the engineering or technical perspective emphasized the importance of involving specialist retailers and technology partners. Furthermore, the political involvement and collaborations with specialized foundations, such as “Swiss Paraplegic Foundation,” were seen as a facilitating aspect to the robotic arm’s implementation. The collaboration with already existing applications was seen as enhancing; however, reliance on third-party providers for services such as “servers and applications” was also identified as a barrier.

##### Policies and Laws

Participants identified various legal barriers to the implementation of the robotic arm, including authorization processes and compliance with European regulations. Stakeholders emphasized the need for compliance with Swiss laws, particularly regarding data protection and the use of integrated cameras in public spaces. Additional concerns arising with the assumption of costs by the Swiss Invalidity Insurance (IV) were noted as barriers to the robotic arm’s implementation.

##### Financing

During the focus group interviews, potential funding sources discussed included the IV, the Swiss National Accident Insurance Fund (SUVA), indemnity insurance, and foundations. Concerns were raised that the robotic arm might cover activities already provided with other ATs, reducing its relevance and likelihood of financing. The reallocation of resources from other services, if IV covered costs for items such as “door openers,” was seen as another barrier, as it could potentially limit the autonomy of the affected persons. The absence of a specific category for robotics in IV regulations posed a barrier to financing, despite “federal court judgments” indicating otherwise. Conflicting motives between cost bearers and developers were further identified as a barrier to financing efforts. Establishing a clear application area for the robotic arm was deemed necessary but challenging, although a “step-by-step approach” was identified as a facilitating factor.

##### External Pressure

Participants noted the duplicating and commercializing of prototypes as barriers. The existing competition from robots, such as “comparable robot arm Lio” in the Swiss market, was cited as a barrier, while Switzerland’s small market size posed challenges in effectively meeting demand.

#### Domain III: Inner Setting

##### Structural Characteristics

The range of the field of application of the robotic arm from personal household of the user to public space was seen as a facilitator by the participants. However, the individuality of the user and the environmental settings also represented a barrier, where, for example, the variety of door handles could be limiting. Furthermore, the discrepancy between indoor and outdoor settings was perceived as a barrier.

##### Relational Connections

The enhancement of autonomy by the robotic arm was perceived to have a positive influence on the users’ state of mind, reducing tension in the immediate vicinity and improving relationship quality.

##### Tension for Change

The “reduction of shortage of skilled labor” within the Swiss care setting was perceived as a facilitator to the implementation of the robotic arm.

#### Domain IV: Individual Domain

##### Opinion Leaders

The current “wheelchair-bound Swiss National Councilor” was mentioned as a facilitator by an engineering stakeholder.

##### Innovation Deliverers

Physical and occupational therapists, as well as specialist retailer and distribution centers, were identified by the participants as innovation deliverers. Here, “Swiss Med Tech, Ortho Reha Suisse, Otto Bock, and the United States Military” were mentioned to enrich the project with expertise and possible resources.

##### Innovation Recipients

Persons with tetraplegia were identified as innovation recipients, where living independently was seen as a facilitator, while for users requiring 24-hour care, such as those dependent on a tracheostomy, the robotic arm was considered unsuitable.

##### Characteristics Subdomain: Need

The “desire for independence” was seen as the main need for the innovation across all focus group interviews. The fact that the robotic arm enabled additional autonomous activities was seen as a facilitator by the participants. Activities such as applying makeup, “eating meals while still warm,” “scratching the face,” and “blowing the nose” were mentioned. In addition, dressing, shopping, and pursuing hobbies such as “art” and “playing games” were cited. However, with additional functions, safety concerns were emphasized. Furthermore, it was seen as a barrier that other supplementary services would still be required and should not be excluded for the user when using the robotic arm.

### Implementation Outlook

The implementation outlook was structured on the fifth domain of the CFIR (implementation process) displayed in Table 2; the included data came from literature research. The formation of an experienced implementation team is proposed to lead the implementation [40], while the needs for ATs for individuals with tetraplegia have been identified in a cross-sectional study (N Hutmacher, unpublished data, March 2025). As the project progresses, insights will be mapped to CFIR constructs, and an implementation plan will be developed, including pilot testing. The use of hybrid type 1 and type 2 designs will allow to assess both effectiveness and implementation within real-world contexts [53].

**Table 2:** Implementation outlook mapped on the fifth Consolidated Framework for Implementation Research (CFIR) domain (constructs with no barriers or facilitators were omitted).

Constructs			Propositions
**Domain V: Implementation process**			
	Teaming	An implementation team with experienced representatives from Switzerland [37]	
	Assessing needs	A cross-sectional study assessing the needs for assistive technologies for individuals with tetraplegia (N Hutmacher, unpublished data, March 2025) Stakeholder representatives from the focus groups for ongoing dialogue and refinement of understanding the needs of both innovation deliverers and recipients Stakeholder groups identified in the focus group interviews to ensure comprehensive understanding of needs	
	Planning	With project advances, mapping of further insights on context with identified and omitted CFIR constructs The rating of identified constructs within the CFIR reflects both their valence and magnitude [54] An implementation plan developed by the implementation team, including specific steps such as pilot testing and measures Use of hybrid type 1 design (effectiveness within real-world contexts), preceded by a hybrid type 2 design [53]	
	Engaging	Possible key players for engagement: the SCI^a^ community in Switzerland and the Swiss Paraplegic Centre and its foundation (national and international scope) [49,55]	
	Reflecting and evaluating: implementation outcomes	Combination of qualitative and quantitative measurements Fidelity: fidelity of implementation assessment and stages of implementation completion [53], including self-reporting Feasibility: surveys Acceptability: surveys and interviews Appropriateness: surveys and focus groups Sustainability: sustainment measurement system and RE-AIM^b^ framework [52,53] Readiness for innovation and implementation: stages of implementation completion tool (postimplementation sustainment outcomes [53]	
	Reflecting and evaluating: innovation outcomes	Combination of qualitative and quantitative measurements Innovation effectiveness: used cases and controlled trial design Functional independence for ADLs^c^: Functional Independence Measure Quality of life: Short Form 36 Health Survey not only for users but also caregivers and ISCoS^d^ quality of life questionnaire [54] User satisfaction: Quebec User Evaluation of Satisfaction with Assistive Technology [56] Specific function outcomes: mobility and communication subscores of Spinal Independence Measure [57]	

^a^SCI: spinal cord injury.

^b^RE-AIM: Reach, Efficacy, Adoption, Implementation, and Maintenance.

^c^ADLs: activities of daily living.

^d^ISCoS: International Spinal Cord Society.

## Discussion

This study aimed to explore the research translation gap in integrating ATs into social and health insurance systems. It specifically focused on identifying stakeholders, assessing contextual factors, and proposing an implementation outlook for introducing an assistive robotic arm into the everyday private lives of individuals with tetraplegia in Switzerland.

### Stakeholders

Throughout all focus group interviews, there was agreement that the present stakeholder groups were relevant to the implementation of the robotic arm. A stakeholder group that was frequently discussed among the interviews was individuals providing a political perspective, which is also emphasized by Wolfenden et al [54]. According to a recent study by MacLachlan et al [56], AT policy should be grounded in evidence, but its foundational framework must go beyond rigid scientific criteria and be more inclusive. This is an important consideration for governments because the capacity to implement can be hindered by the volume of concurrent initiatives, potentially leading to low prioritization of the implementation, which could cause the implementation to fail [57]. In this context, stakeholder perspectives, contextual factors, cultural nuances, available resources, and systemic viewpoints should be systematically evaluated and synthesized in a transparent manner to enhance their credibility and inform policy decisions. MacLachlan et al [56] even propose the role of policy entrepreneurs, translating technical content into compelling information to engage politicians, to network and interact with the key stakeholders. Involving the current wheelchair-bound Swiss national councilor, as discussed in the focus group interviews, could facilitate cross-sector collaboration and have positive effects on the implementation of the robotic arm [58].

### Barriers and Facilitators

All stakeholder groups agreed that the innovation addresses the needs of the recipients, specifically the desire for greater autonomy among individuals with tetraplegia. The robotic arm would allow for improvement in daily life participation, and although some activities may still be challenging or time consuming [59], addressing the impairment of the upper extremities and the autonomy provided was valued by the users [7]. Because the robotic arm may not allow complete independence for affected persons, partial or continuous assistance from a caregiver might still be needed, which was perceived as negative in the focus group interviews. Similar results were found by Gelderblom et al [24] assessing the MANUS robotic arm, where the continued dependence on caregivers was perceived as stressful to the user. Nevertheless, according to the focus group interviews, users’ quality of life was expected to improve with the use of the robotic arm. This is reflected in the observations of Maheu et al [28], where the empowerment of the user enables an improvement in the quality of life, not only of the user but also of the caregiver. This facilitator must be emphasized as an important contribution of the robotic arm [60]. Specific assessment of how ATs, more precisely robotic arms, influences the quality of life of affected persons and of caregiver needs to be addressed in future research.

The complexity of the requirements of the innovation and the need for individuality with each user were perceived as barriers to the implementation of the robotic arm. The involvement of users in the development and implementation process was emphasized across the focus group interviews. This not only provides valuable insights that can enhance the effectiveness of the innovation [61,62] but also leads to higher user satisfaction. According to Martin et al [63], users that possess a greater understanding of ATs and feel more informed tend to exhibit higher satisfaction with the devices. Likewise, when their requirements are unmet, the users tend to express less satisfaction levels with the technology. It is anticipated that if usability limitations, such as use efficiency and overall satisfaction, remain unaddressed, users are prone to abandon their AT shortly after acquiring it [30,64]. This is an important factor to consider when assessing the readiness of the AT market and the implementation of the robotic arm moving forward. Furthermore, piloting, as mentioned in the focus group interviews, would allow users to gain experience and further adapt the innovation based on their feedback [65].

According to Nicolet et al [66], it is crucial to involve not only users but also other diverse stakeholders in the early stages. This facilitates the dissemination and transfer of findings, while also enhancing credibility and acceptance within the population. This is relevant as the implementation of the robotic arm could be further facilitated by support from the population. The general positive attitude among the focus groups toward technology as the future presents an optimal prerequisite for this aspect. The favorable view of robots was also shown among the older adult population in Switzerland, although concerns about personal connection and data privacy were raised, supporting similar statements from the focus groups [67]. Understanding patient needs and resources and effectively prioritizing them in combination with the involvement of the public, strongly correlate with successful implementation and are seen as a way to secure the acceptance and implementation of policies [57,68]. These are relevant aspects to consider as we move forward with the implementation of AT, specifically the robotic arm.

A relevant barrier discussed across all the focus group interviews was the financing of the robotic arm. Its current costs as a prototype exceed individual financial resources of affected persons, making it unaffordable. In Switzerland, the governmental social or health insurance system is expected to reimburse costs for ATs. The primary focus of social insurers lies in the cost-effectiveness of the assistance they are considering reimbursing, which according to Romer et al [69] specifically refers to the extent of cost savings achievable through the acquisition of the AT in relation to the overall cost of care for the user (cost of labor of the caregiver and cost of technical aids), which the robotic arm could replace. Additional indirect economic advances of the robotic arm encompass various benefits, such as reductions in unemployment compensation expenses, as users regain employment opportunities or increased workload can be allowed or area of application of work be expanded [69]. According to Bensi et al [56], cost savings of €290 (US $319) per individual over a span of 5 years resulted from enhanced autonomy through ATs and decreased reliance on personal assistants. The delicate balance between affordable supply of needed assistive products and high quality to foster social benefits alongside financial profitability remains a challenge. However, evidence on the effectiveness and cost-effectiveness of assistive robotic arms is limited [70], necessitating further research contributing to the implementation process [71].

In addition, determining the cost bearer within Switzerland’s social insurance system is crucial for the implementation of ATs and was mentioned as a barrier across the focus group interviews. Laws in Switzerland require the IV to provide rehabilitation services, while the SUVA covers the economic consequences of accidents or occupational diseases [72,73]. The complexity and fragmentation of the Swiss social insurance system [74] could hinder the implementation process. This is supported by Schusselé Filliettaz et al [75], who found in a survey on integrated care initiatives in Switzerland between 2015 and 2016 that chronic care integration presents a notable challenge due to issues with the allocation of responsibilities, payment structures for health care professionals and institutions, and financing mechanisms. This is further complicated by the different conditions that apply to the assumption of costs by the various cost bearers within the social insurance system. The assumption of costs by the IV, which was emphasized in the interviews, requires the robotic arm to fulfill the criteria effectiveness, appropriateness, and economic efficiency [76]. In addition, the List of Remedies and Equipment (MiGeL) outlines the device categories eligible for coverage and specifies the maximum reimbursement amounts, including approximately 20 groups of approved devices [77]. Currently, no category that includes robotics as an AT is listed in the MiGeL. Without a designated category, similar ATs listed on the MiGeL may receive compensation, potentially leading to higher costs or inadequate support compared to the robotic arm. According to MacLachlan et al [78], the absence of a clear framework for ATs such as robotics hinders implementation efforts, further compounded by a lack of familiarity and knowledge regarding disability and impairment. The clarification of competence and the assumption of costs among the various cost bearers need to be addressed for AT and the robotic arm in the Swiss context.

Moving forward with the implementation of the robotic arm, a focused approach targeting the identified barriers is necessary. The key recommendations from the authors for addressing the most relevant barriers currently identified are listed below:

- Conduct a detailed cost-effectiveness study to demonstrate the robotic arm’s long-term financial savings (e.g., reduction of reliance on personal assistance, improvement of workability, and saving unemployment benefits).
- Perform pilot studies, where individuals with tetraplegia can trial the robotic arm in real-world settings. Continuous feedback should be collected to identify potential issues with usability, fit, and comfort. Furthermore, caregivers should be involved in the trial process of the robotic arm. This will help to construct the robotic arm to meet the user’s needs and make it more effective.
- Clarify cost assumptions with insurers and enter into dialogue with the IV, SUVA, and other relevant insurance bodies to establish clear guidelines for the reimbursement of the robotic arm and comparable ATs. Here, the demonstration of the robotic arm’s cost-effectiveness will help justify reimbursement.

### Implementation Outlook

Moving forward, an implementation team overseeing and facilitating key activities in the selection and implementation is needed [79]. Representatives of implementation teams should encompass various viewpoints, including those of practitioners, supervisors, administrative leaders, and policy makers. The relevance of implementation teams was also acknowledged by Metz and Bartley [79], stating that in a survey of implementation frameworks, 17 (68%) of 25 frameworks emphasized the formation and use of implementation teams as essential elements of the implementation framework to ensure high-quality implementation. Furthermore, a stakeholder analysis by Reed et al [80] would allow a thorough investigation of relevant stakeholders for overall stakeholder engagement and their change over time [40,81-83] for the implementation of ATs.

In addition to the implementation context, successful evaluation of innovation and implementation is needed. In this research, the combination of innovation and implementation outcomes was given as an implementation outlook, as suggested by Hung et al [84]. According to Albers et al [40], clinical outcomes may be inaccurate or poorly understood if implementation outcomes are not measured, making it crucial to combine both types of assessments. Previous research emphasizes the construct relative advantage of the CFIR as a decisive factor for successful adoption and implementation of the innovation [85]. This suggests that successful implementation is more likely when users recognize a clear advantage in the innovation’s effectiveness. Stakeholders must clearly observe the benefits of the innovation, emphasizing the importance of efforts to demonstrate these benefits [57]. Therefore, giving early attention to the relative advantages in implementation planning could be a strategic approach and should be considered as we move forward with the robotic arm and its implementation.

The use of a hybrid design, combining elements of clinical effectiveness and implementation research to explore not only what works but also how, where, and why, was further proposed in the implementation outlook. This would allow to incorporate complexity to establish external validity and gain insights on scaling up and disseminating [86]. The use of mixed methods designs would enable exploratory and confirmatory research, according to Palinkas et al [87], incorporating the perspectives of potential consumers, practitioners, and clients. To assess innovation effectiveness, standardized ADLs were suggested in this research. Integrating outcomes with the International Classification of Functioning, Disability, and Health could further refine functional evaluation tasks, ensuring a comprehensive assessment that goes beyond basic activities and allows for better comparison across research groups [88]. These considerations for methodology, including design and data collection techniques, need to be incorporated in the future research on the robotic arm’s effectiveness and implementation and comparable ATs.

### Strength and Limitations

Some limitations should be considered together with the results of this research. First, although efforts were made to include a broad coverage of stakeholder groups, the distribution of representatives varied, potentially introducing bias with certain perspectives dominating the discussion [42]. To decrease this group dynamic bias, sampling procedures were used to group similar perspectives together in focus groups. However, the absence of a family caregiver representative within the nursing or care stakeholder group and representatives from the political perspective group was acknowledged as a limitation, as this may have led to missed important insights. Second, the study focused on implementation within the Swiss social insurance system, limiting its generalizability in terms of governmental financing of the robotic arm and similar ATs. Furthermore, the direct end users of the robotic arm considered in this study are persons with impaired upper extremity due to tetraplegia. However, the target population could be broadened to include individuals with upper extremity impairments caused by congenital conditions, such as cerebral palsy, as well as other medical conditions. Expanding the target group will require further research to address the specific needs and challenges associated with these conditions.

The analysis of the contextual factors was conducted early in the development of the robotic arm. This is a strength, as it provides valuable insights and identified barriers and facilitators that should be considered moving forward. However, further exploration and development may highlight additional aspects influencing context, and identified contextual factors need to be reassessed with the process. Another strength of this research is its potential applicability to future AT implementation processes in Switzerland and countries with comparable social and health insurance systems. The use of the CFIR framework allows for building on these results and integrating findings from other studies, contributing to knowledge about the interaction between innovations and context. Another strength of this study is the contribution of valuable insights to the implementation of ATs, specifically assistive robots.

### Conclusions

This research explored the current contextual factors influencing the implementation of an assistive robotic arm in the everyday lives of individuals with tetraplegia in Switzerland. As an example of AT, the robotic arm offers promising benefits in terms of autonomy and improved participation for users, positively influencing their quality of life. Challenges such as high costs and regulatory complexities regarding cost coverage in its implementation need to be addressed, highlighting the need for a framework for implementing ATs. The need for political lobbying, stakeholder engagement, and user involvement throughout the implementation process were identified to facilitate the widespread adoption of ATs in Switzerland and other countries with comparable social and health insurance systems.

## Appendix

Multimedia Appendix 1 Overview of the robotic arm system.

Multimedia Appendix 2 Semistructured interview guide for focus groups (German).

Multimedia Appendix 3 Stimulus material for robotic arm system.

Multimedia Appendix 4 Illustration map for focus group 1 (German).

Multimedia Appendix 5 Illustration map for focus group 2 (German).

Multimedia Appendix 6 Illustration map for focus group 3 (German).

Multimedia Appendix 7 Illustration map for focus group 1 (English).

Multimedia Appendix 8 Illustration map for focus group 2 (English).

Multimedia Appendix 9 Illustration map for focus group 3 (English).

Multimedia Appendix 10 Example of participant validation of the illustration map in Microsoft Forms (German).

Multimedia Appendix 11 Example of participant validation of the illustration map in Microsoft Forms (English).

Multimedia Appendix 12 Patient information and informed consent form.

Multimedia Appendix 13 Barriers and facilitators identified (constructs with no barriers or facilitators were omitted).

## Abbreviations

ADLs: activities of daily living
AT: assistive technology
CFIR: Consolidated Framework for Implementation Research
FRIEND: Functional Robot with Dexterous Arm and User Friendly Interface for Disabled People
IV: Swiss Invalidity Insurance
MiGeL: List of Remedies and Equipment
SCI: spinal cord injury
SUVA: Swiss National Accident Insurance Fund

## Data Availability

The datasets generated or analyzed during this study are available from the corresponding author on reasonable request.
