# Supplementary figures and images for: Stakeholders and Contextual Factors in the Implementation of Assistive Robotic Arms for Persons With Tetraplegia: Deductive Content Analysis of Focus Group Interviews
Source: JMIR Rehabil Assist Technol. 2025 May 16;12:e65759. doi: 10.2196/65759 (PMC12125562; doi:10.2196/65759)

*
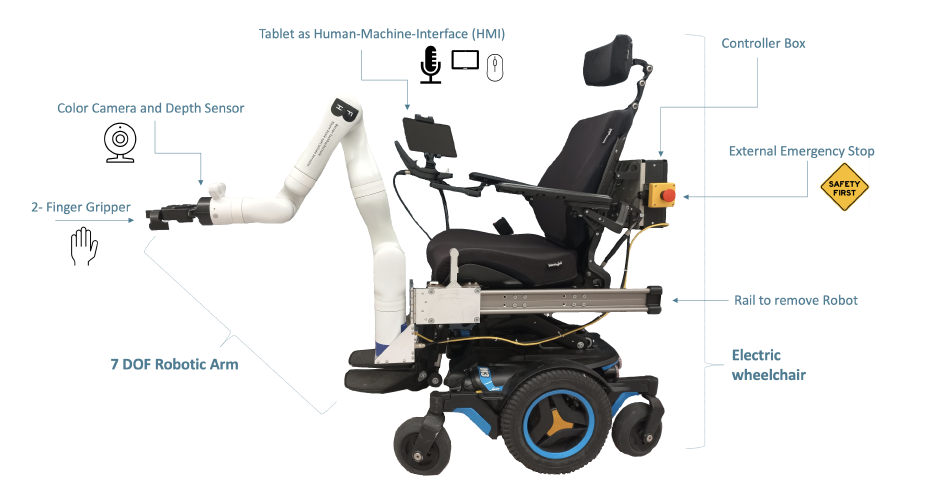
*

Supplement: Multimedia Appendix 1 [file rehab_v12i1e65759_app1.docx]

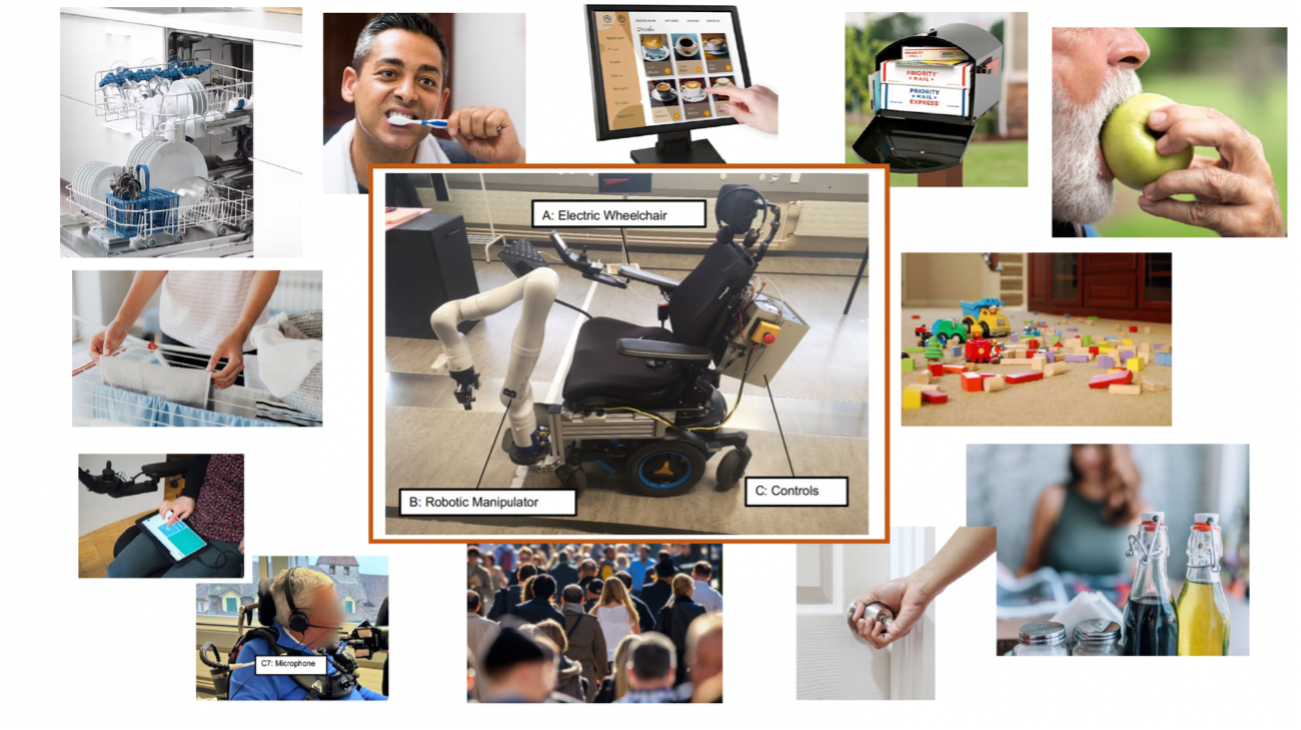

Supplement: Multimedia Appendix 3 [file rehab_v12i1e65759_app3.docx]

## Multimedia Appendix 4 [Illustration Map Focus Group 1 (German)]


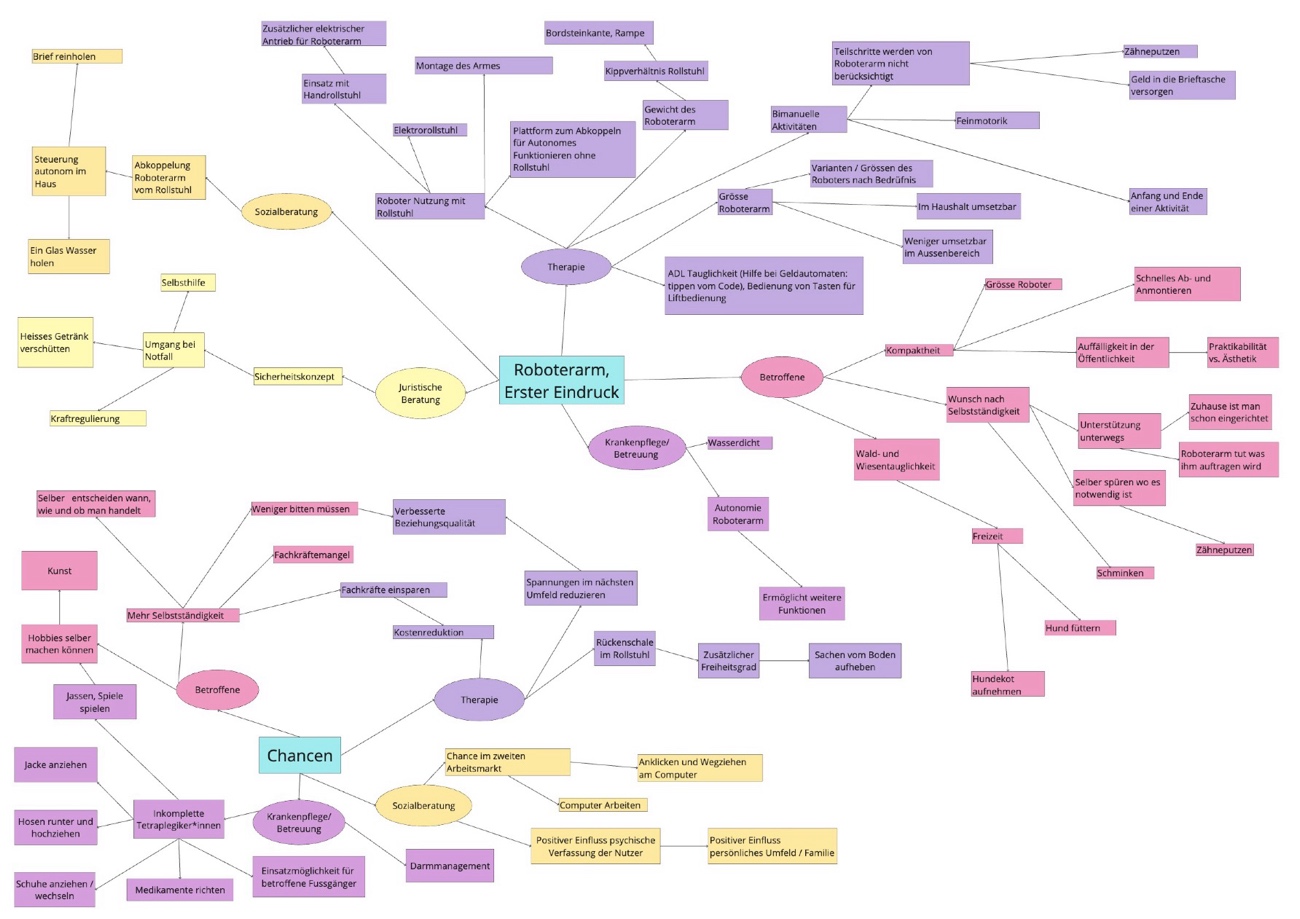

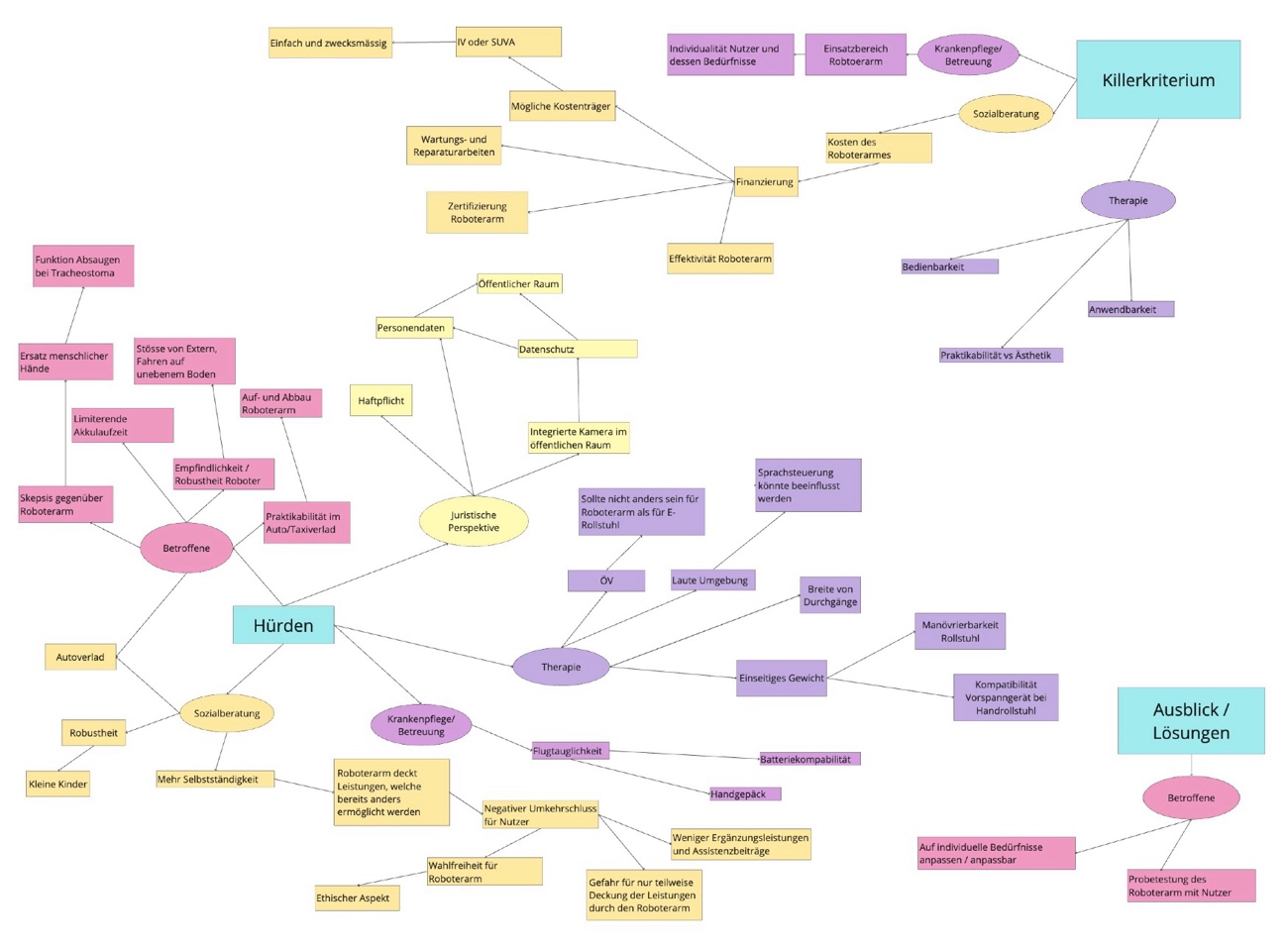

Supplement: Multimedia Appendix 4 [file rehab_v12i1e65759_app4.docx]

## Multimedia Appendix 5 [Illustration Map Focus Group 2 (German)]


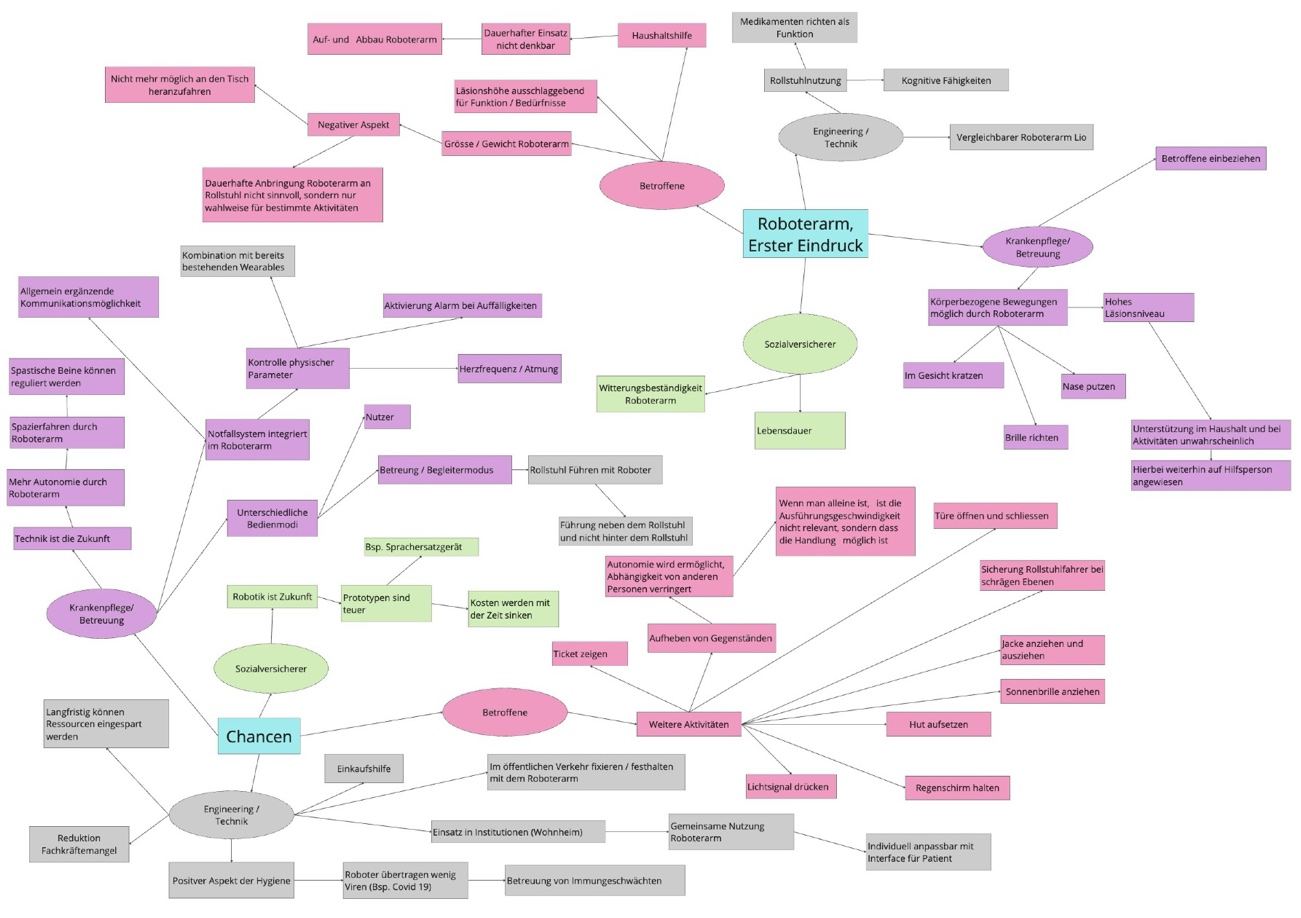


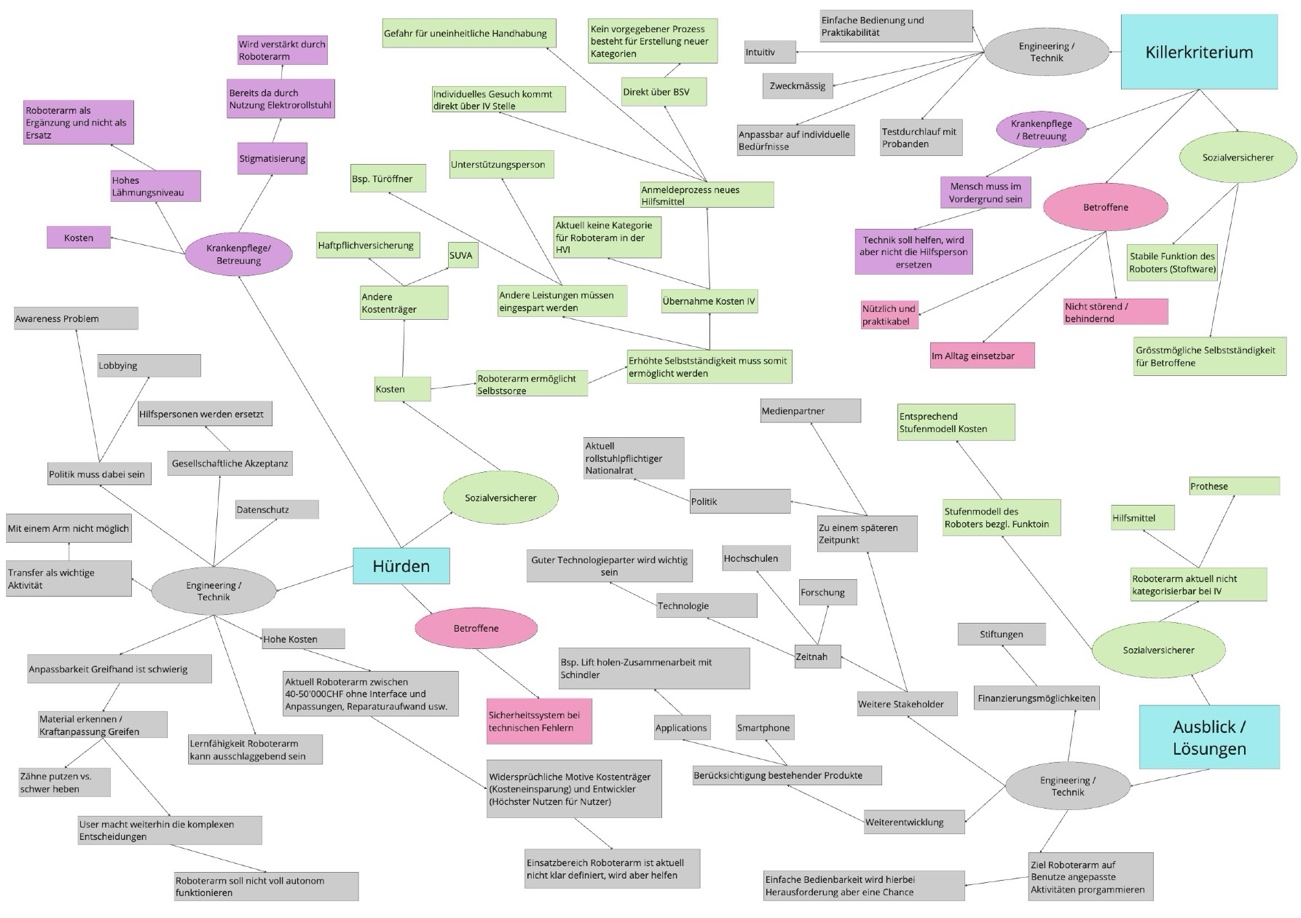

Supplement: Multimedia Appendix 5 [file rehab_v12i1e65759_app5.docx]

## Multimedia Appendix 6 [Illustration Map Focus Group 3 (German)]


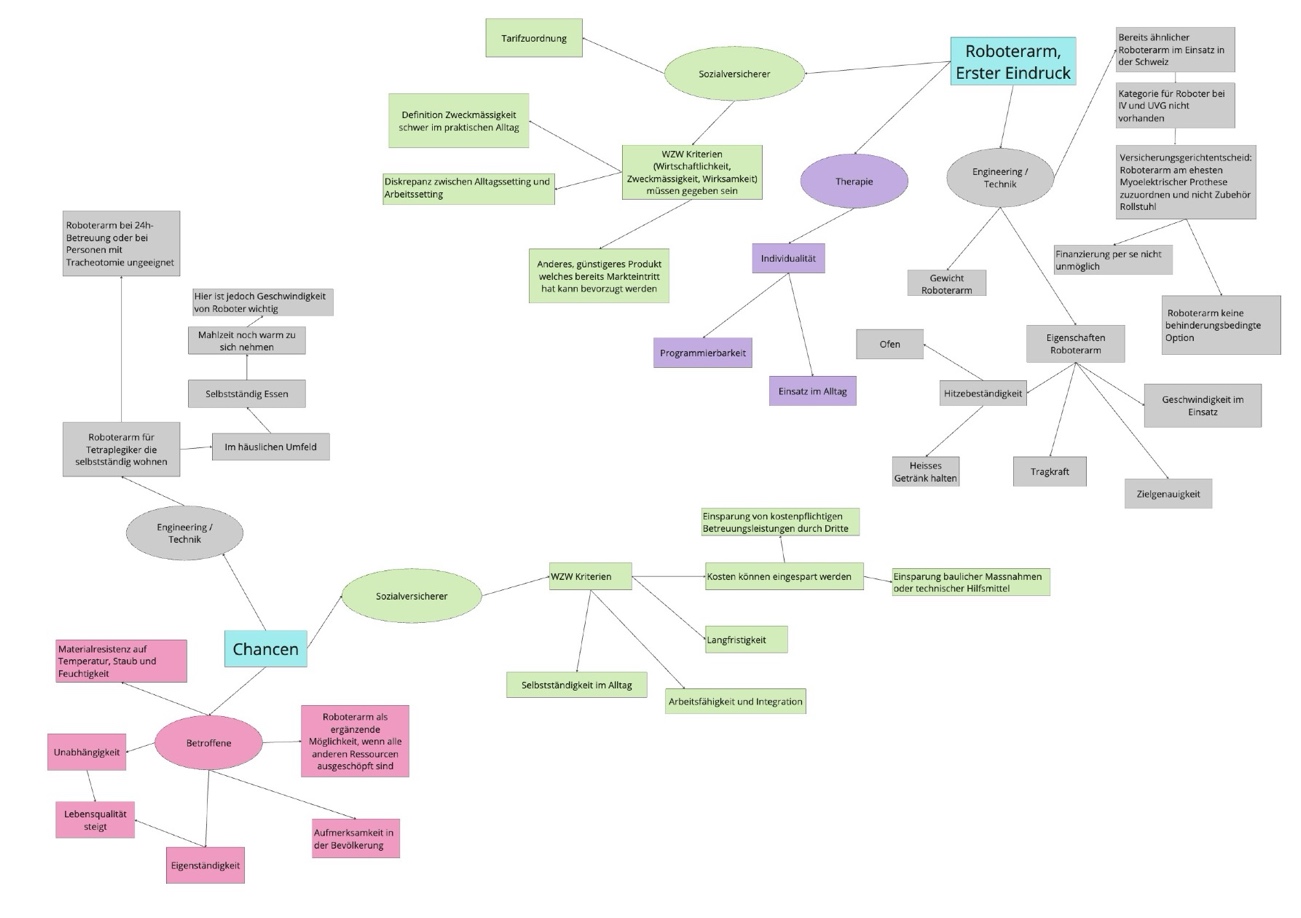


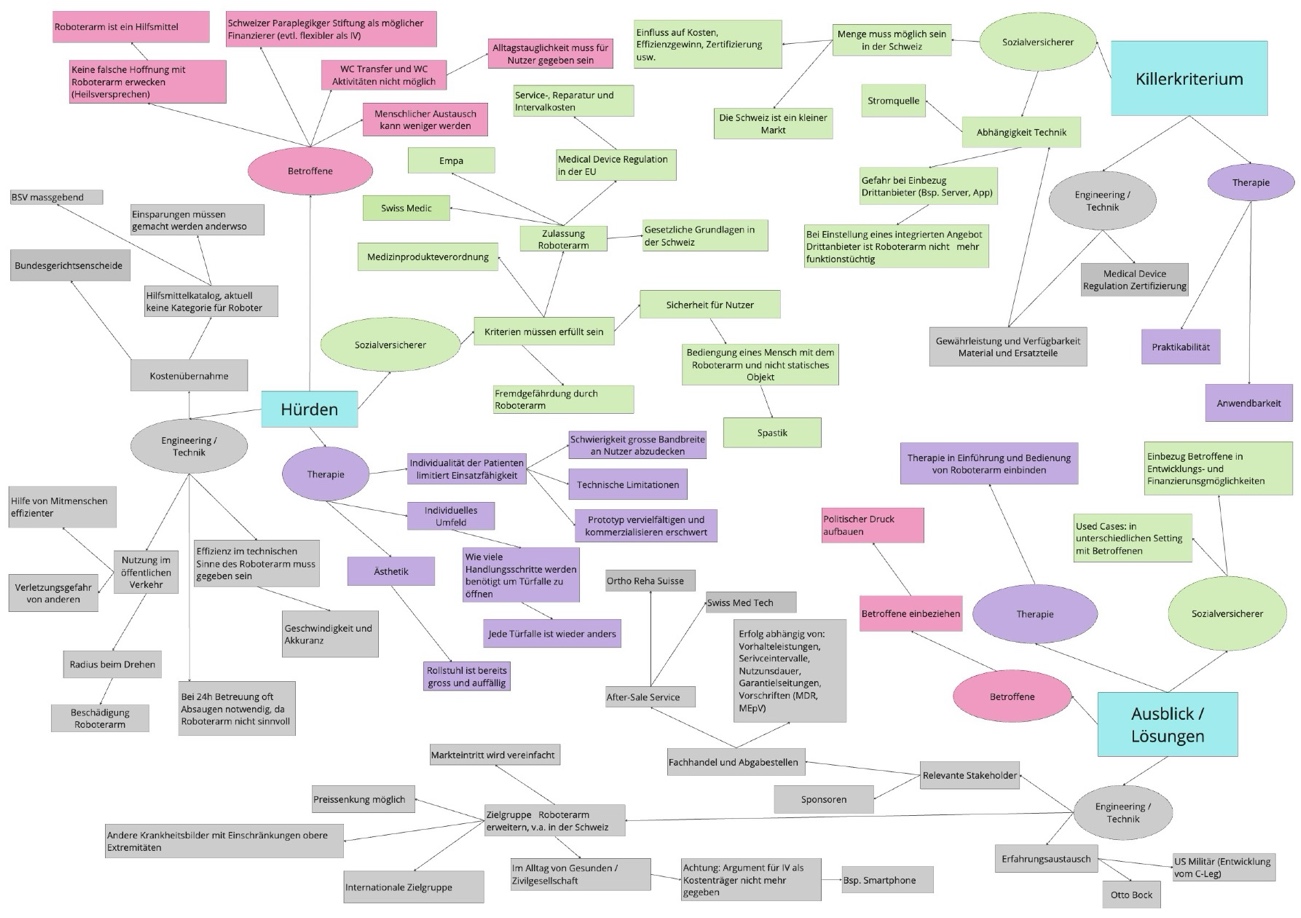

Supplement: Multimedia Appendix 6 [file rehab_v12i1e65759_app6.docx]

## Multimedia Appendix 7 [Illustration Map Focus Group 1 (English)]


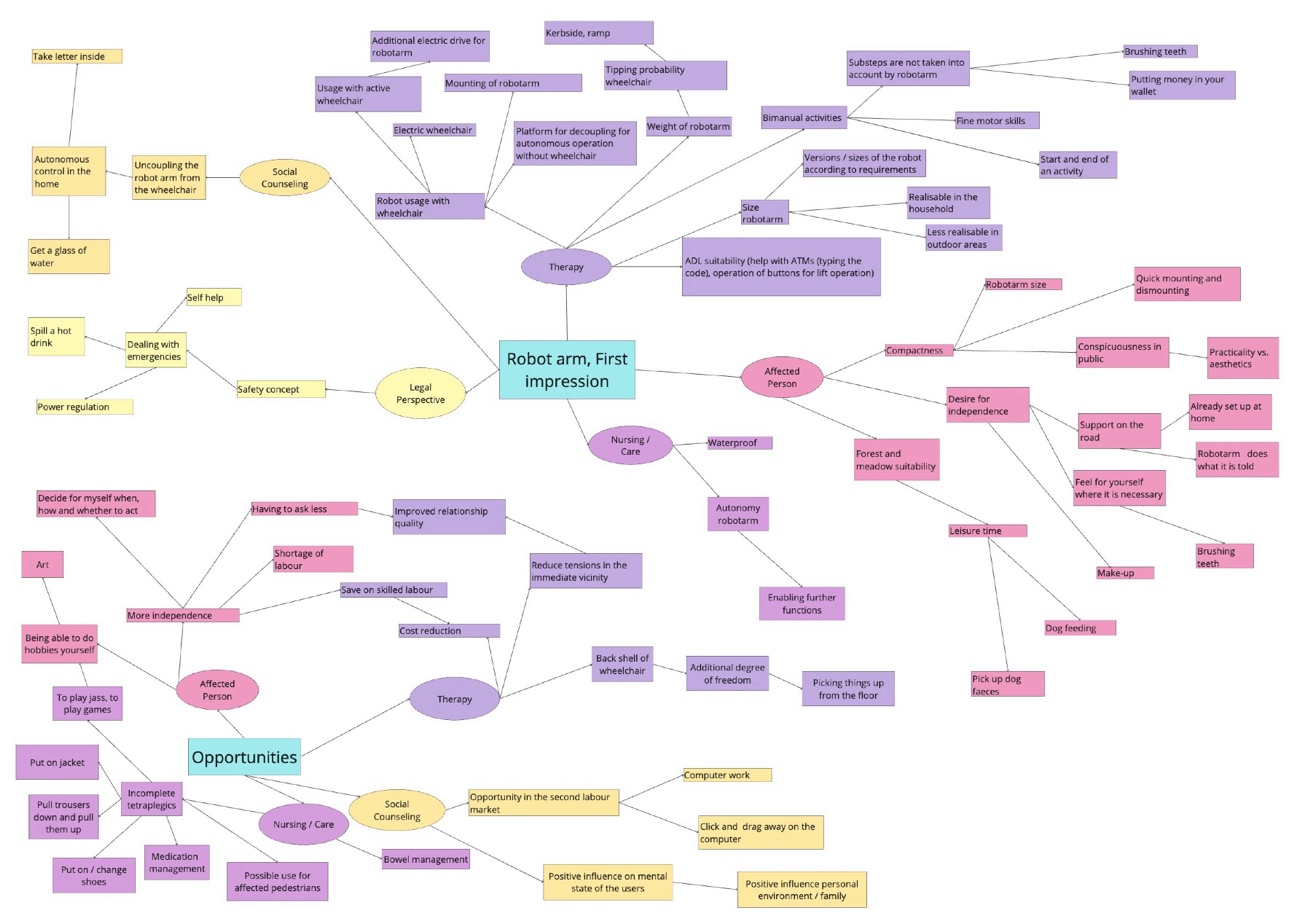


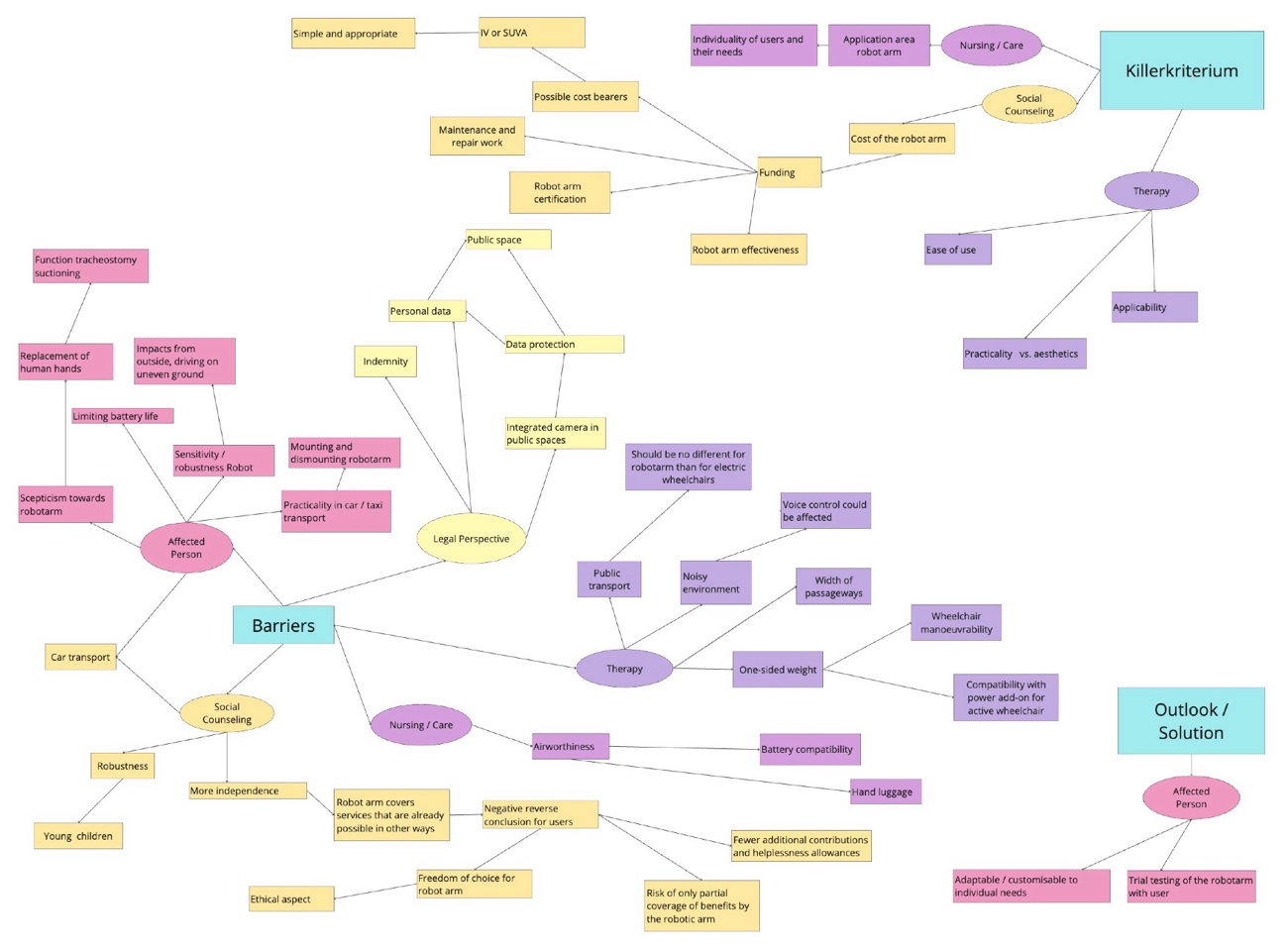

Supplement: Multimedia Appendix 7 [file rehab_v12i1e65759_app7.docx]

## Multimedia Appendix 8 [Illustration Map Focus Group 2 (English)]


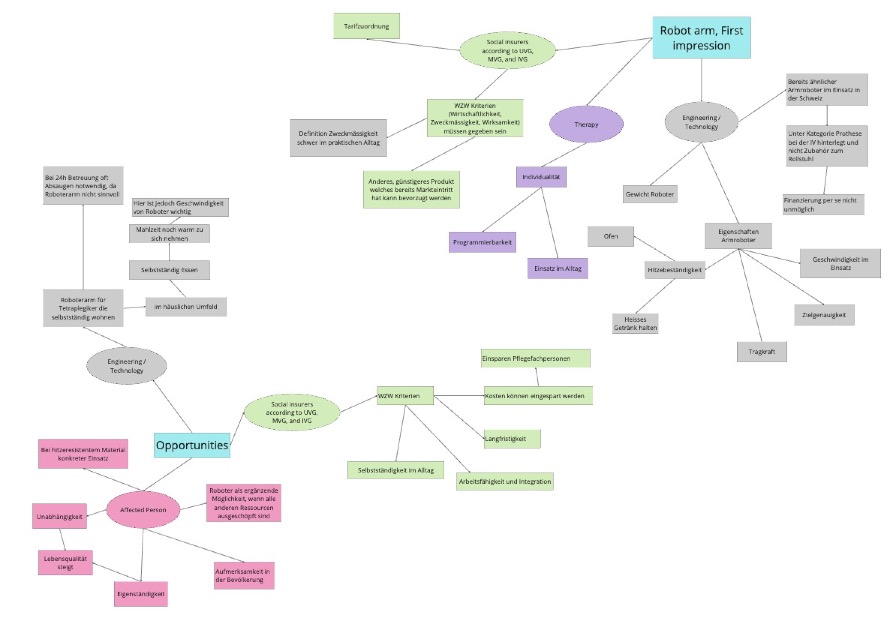


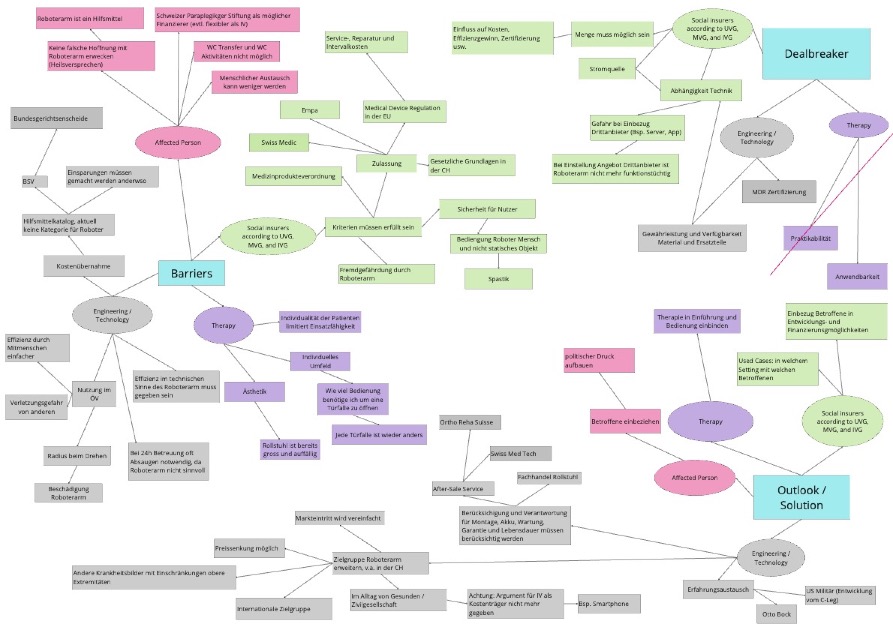

Supplement: Multimedia Appendix 8 [file rehab_v12i1e65759_app8.docx]

## Multimedia Appendix 9 [Illustration Map Focus Group 3 (English)]


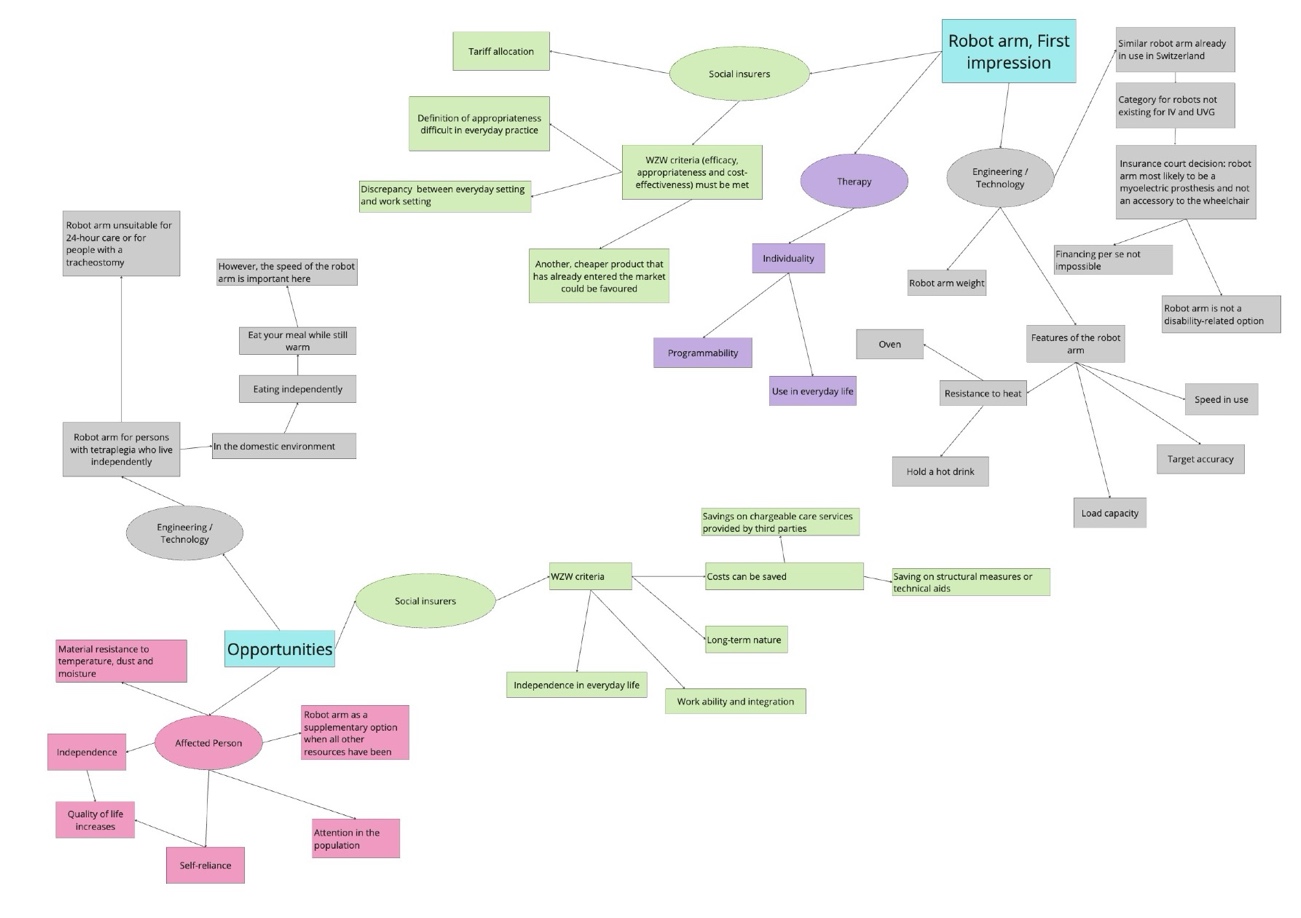


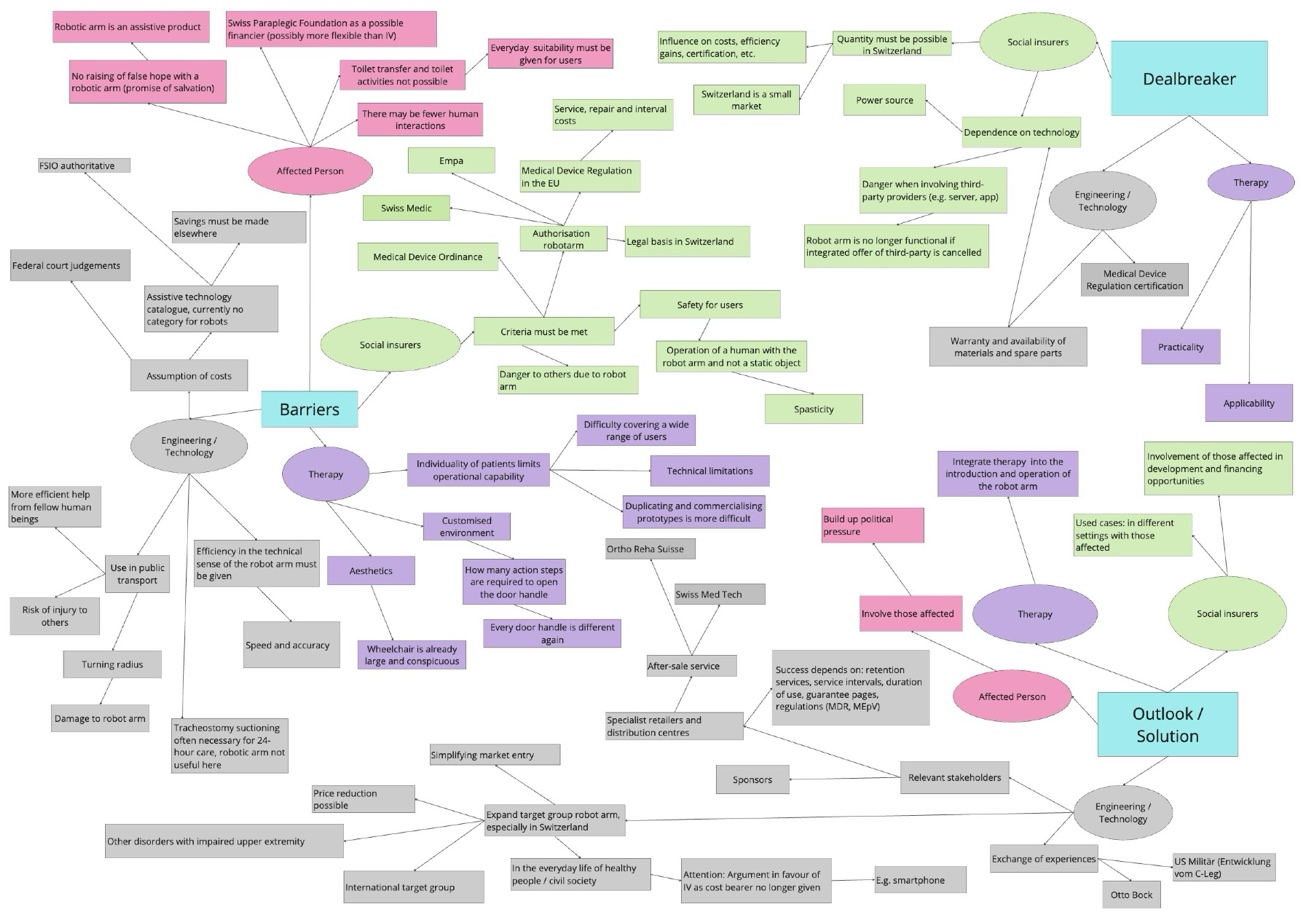

Supplement: Multimedia Appendix 9 [file rehab_v12i1e65759_app9.docx]

## Multimedia Appendix 12 [Patient Information and Informed Consent]


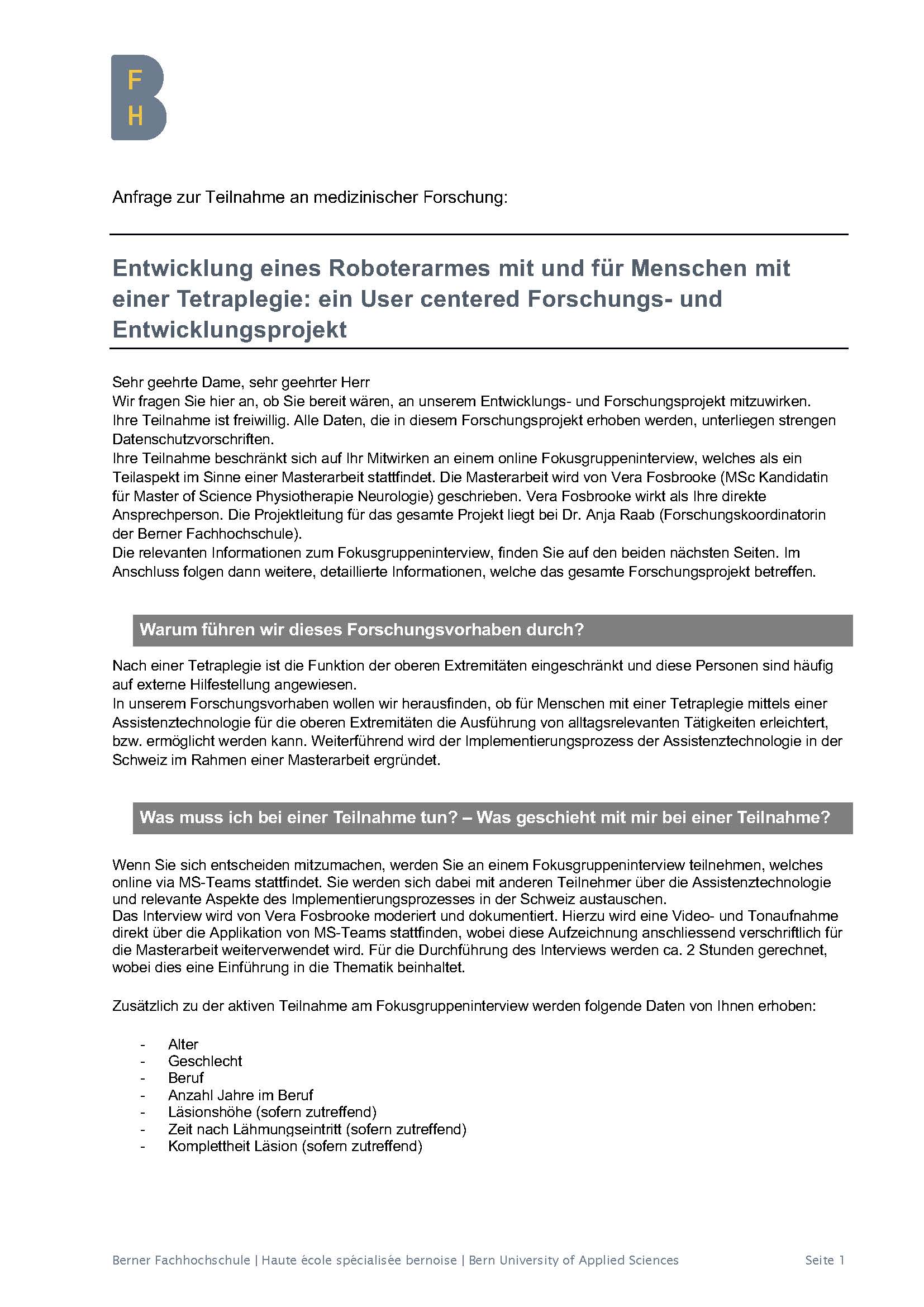


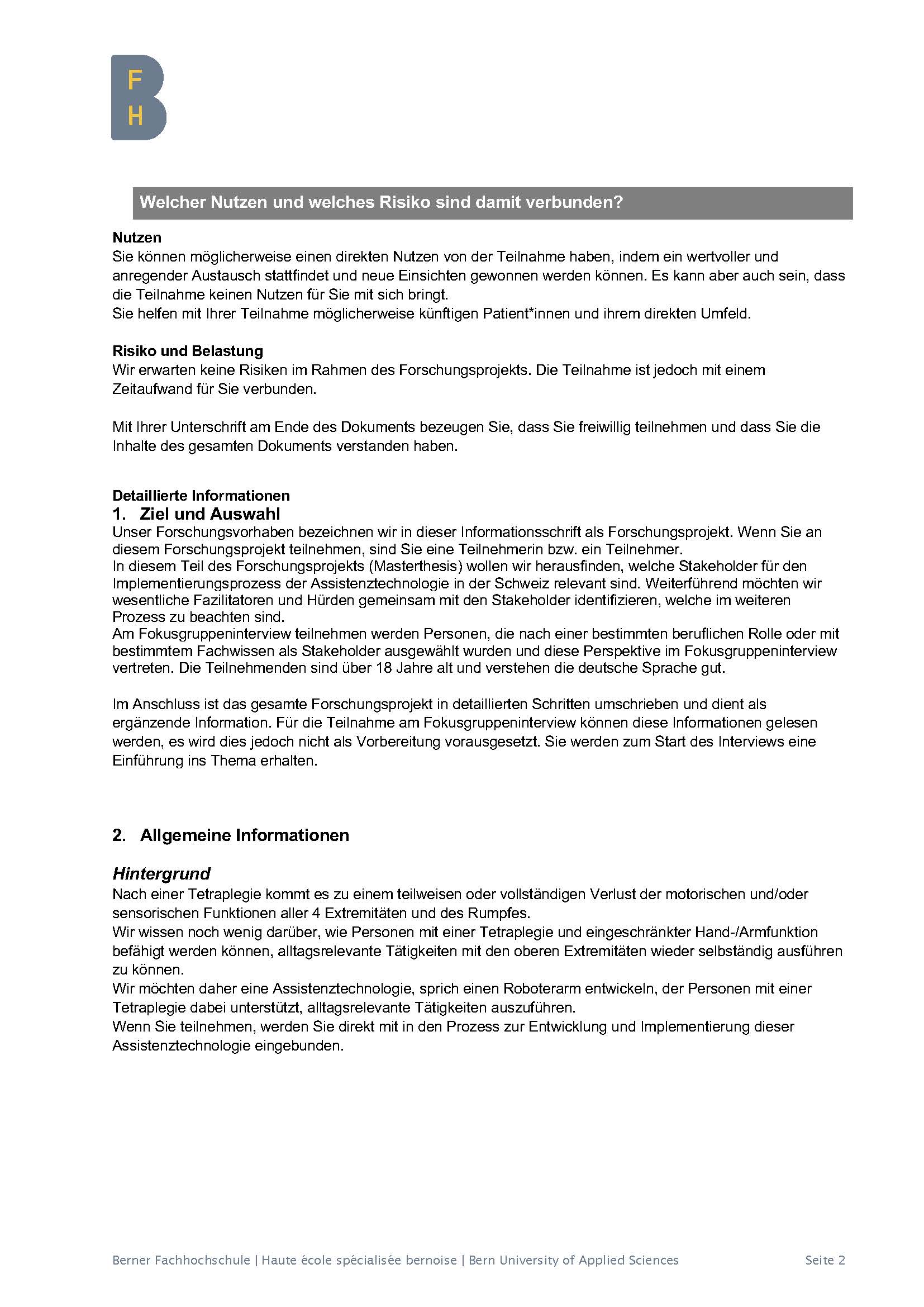

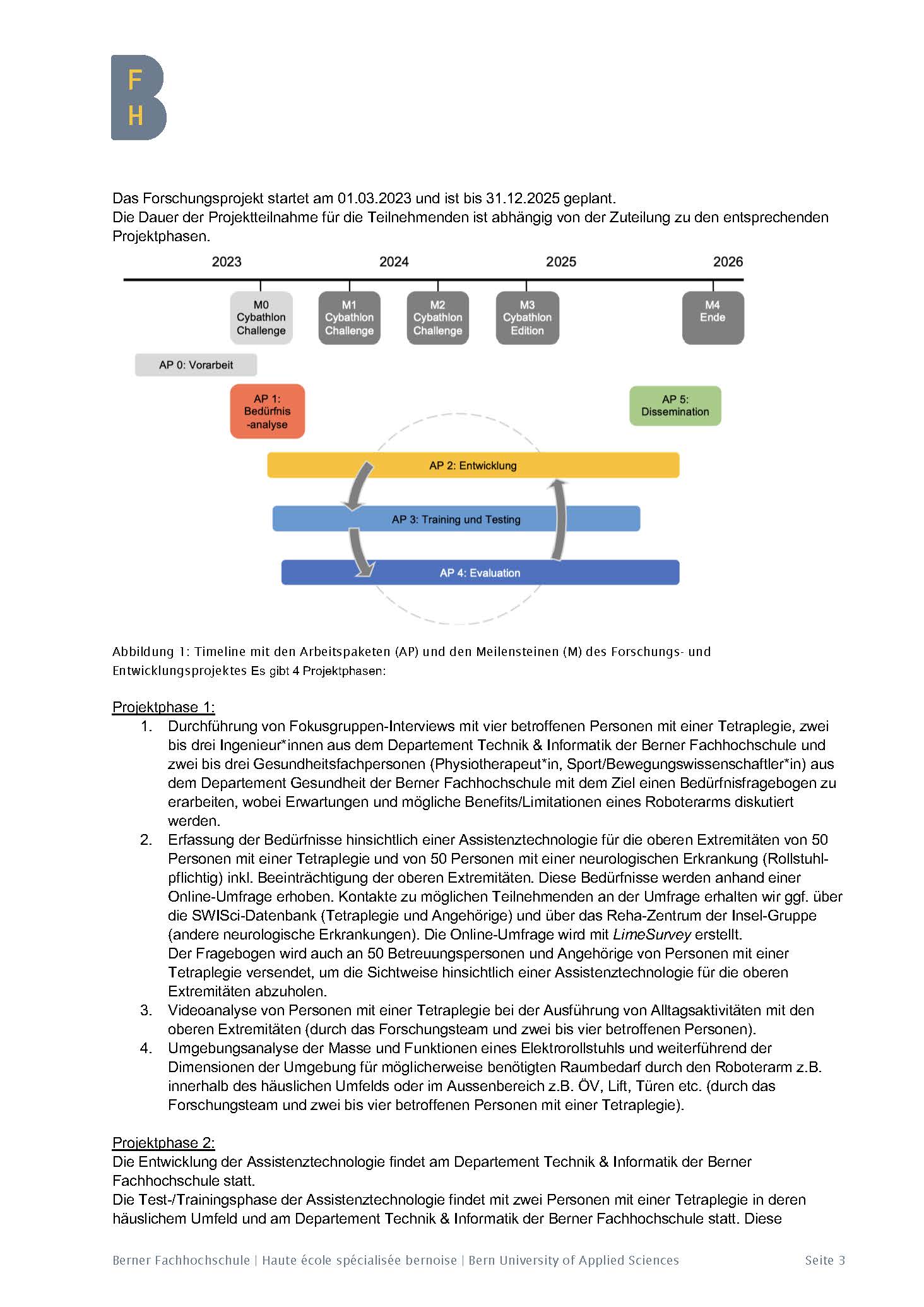


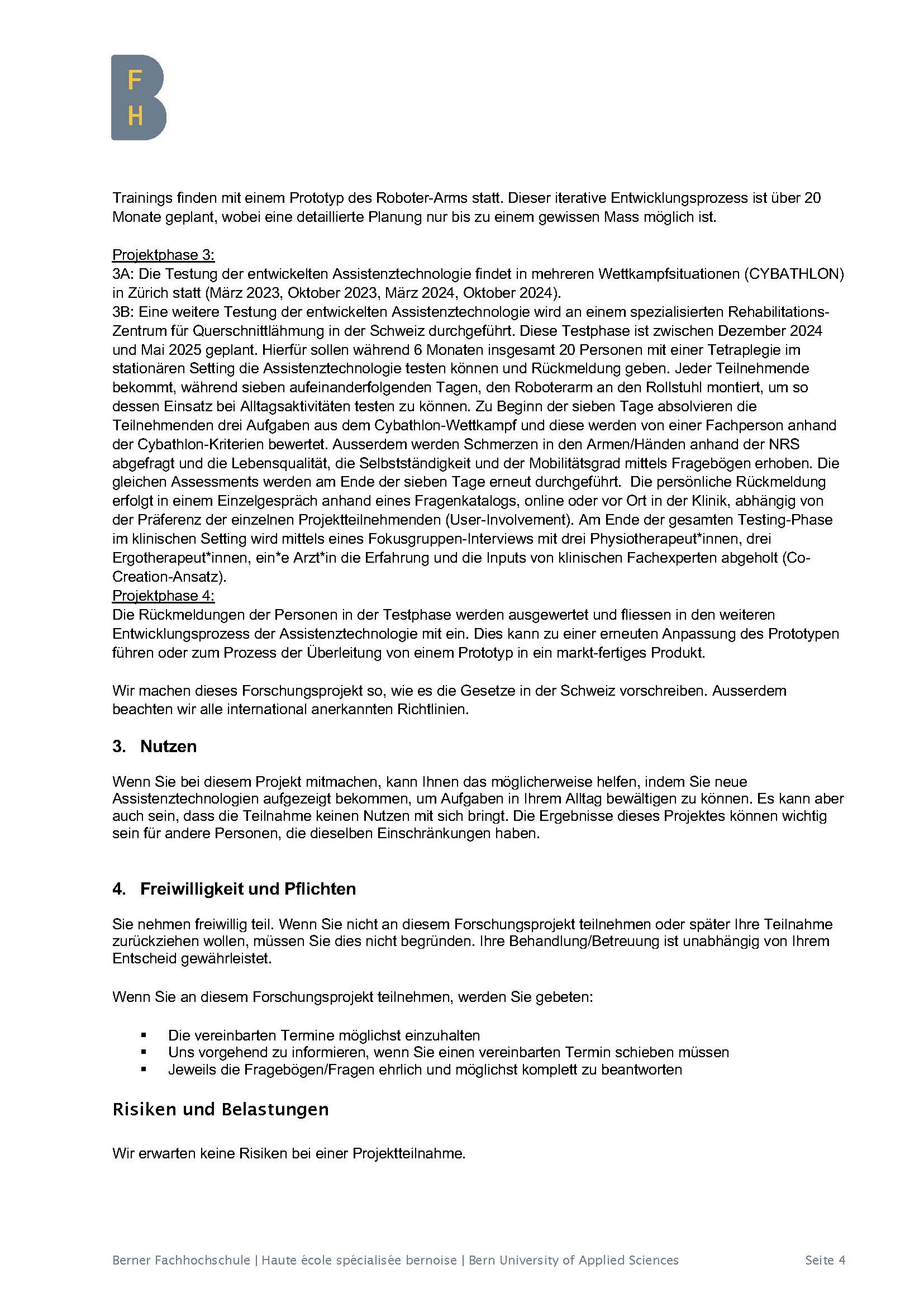

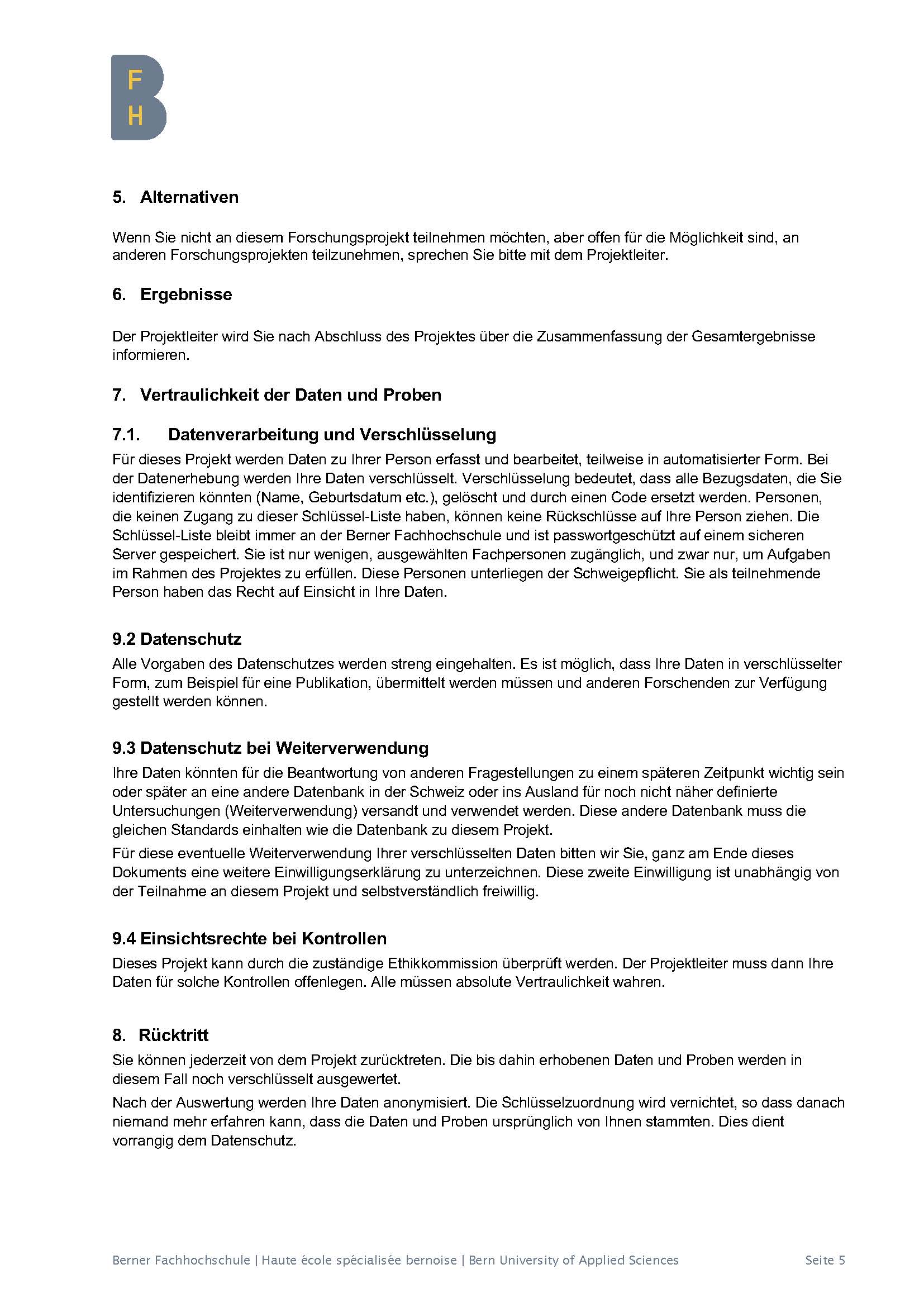


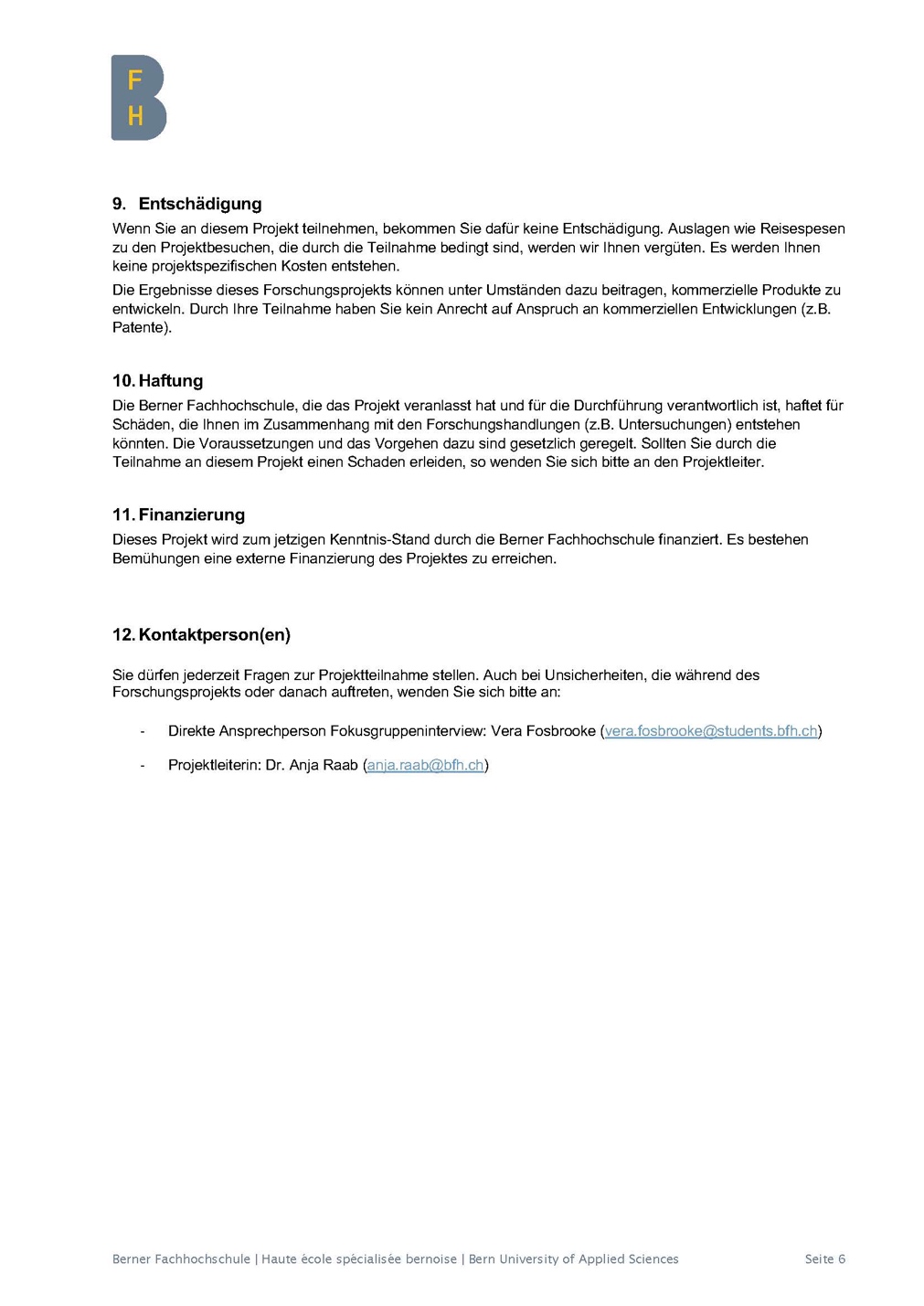

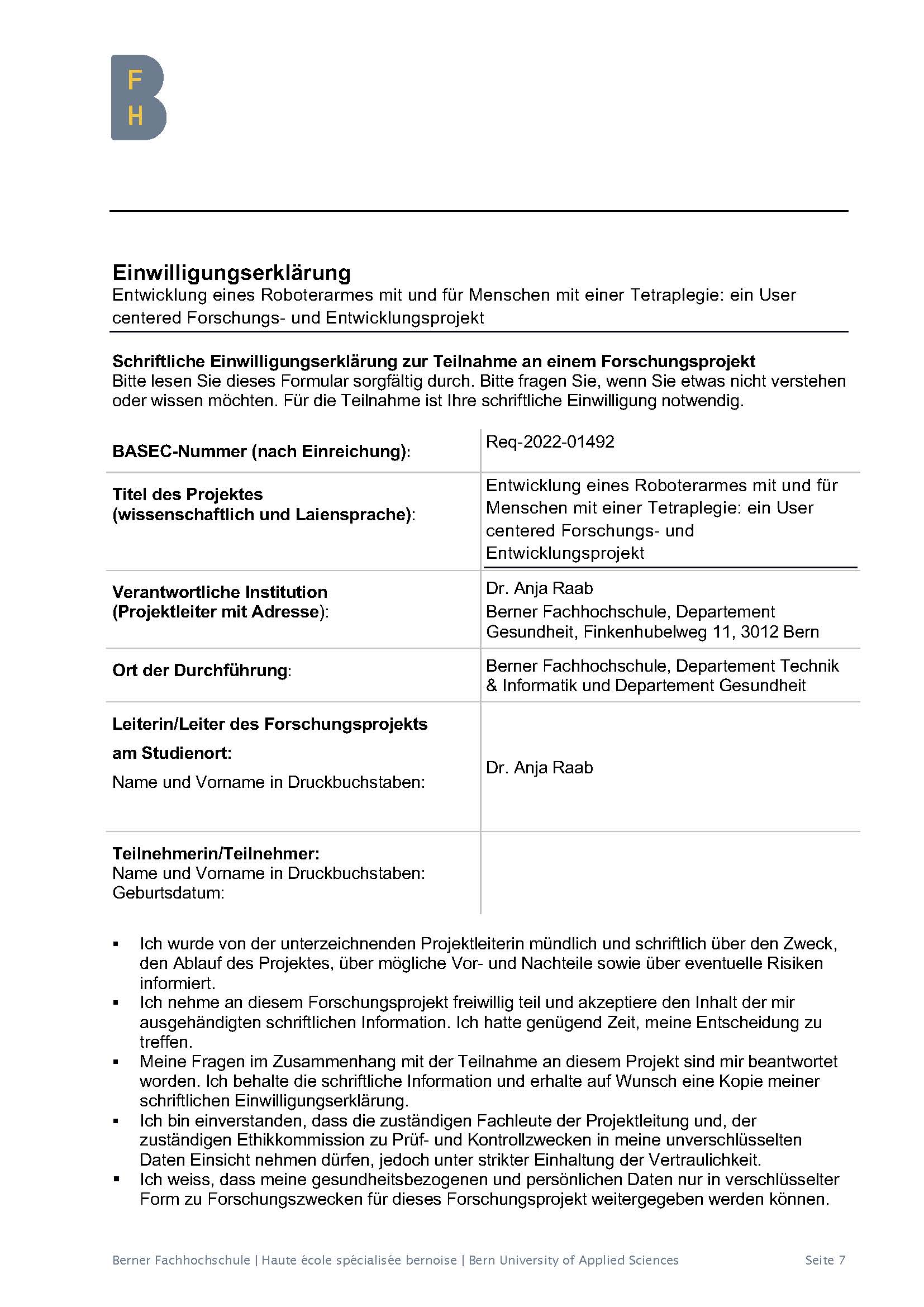


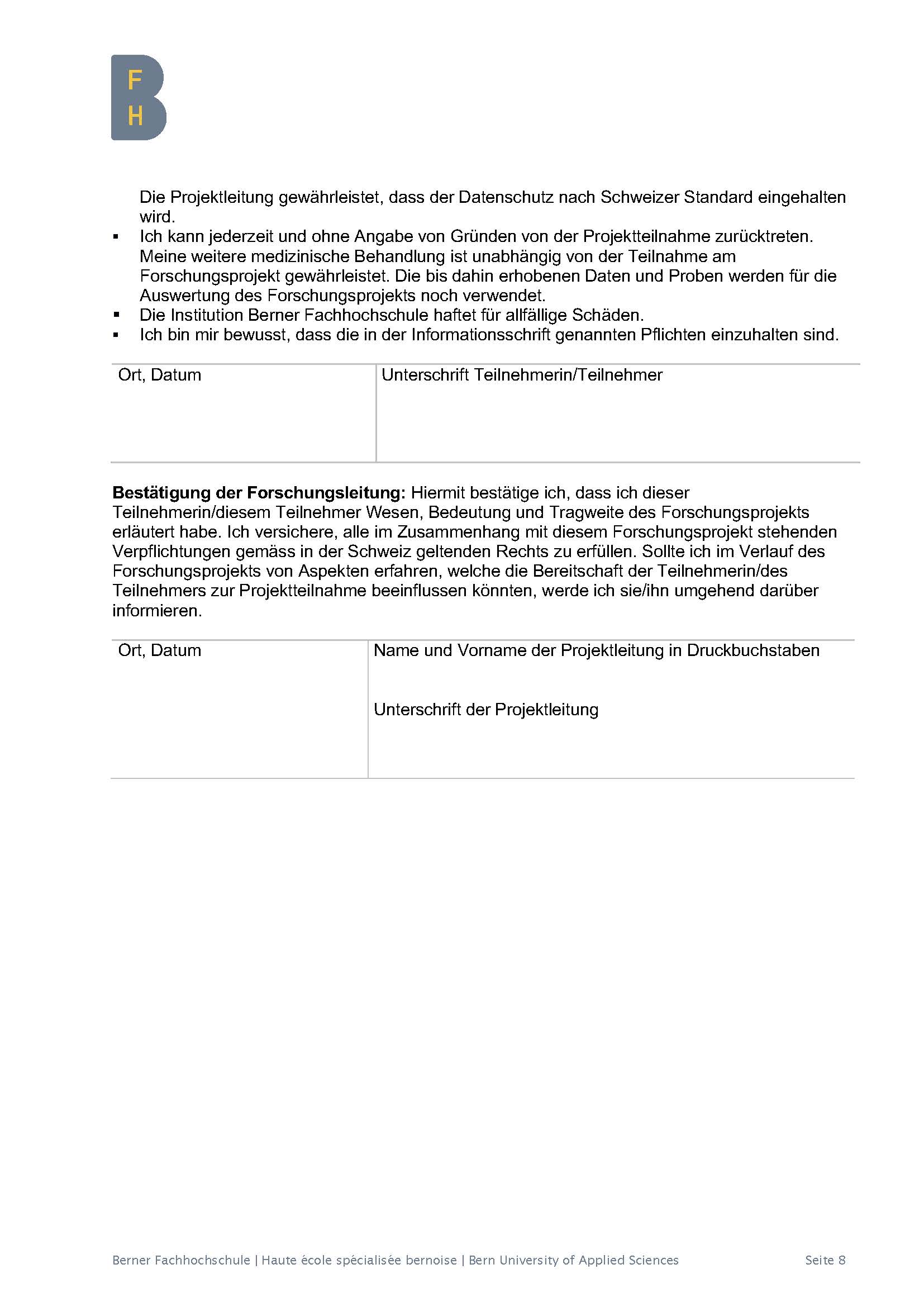

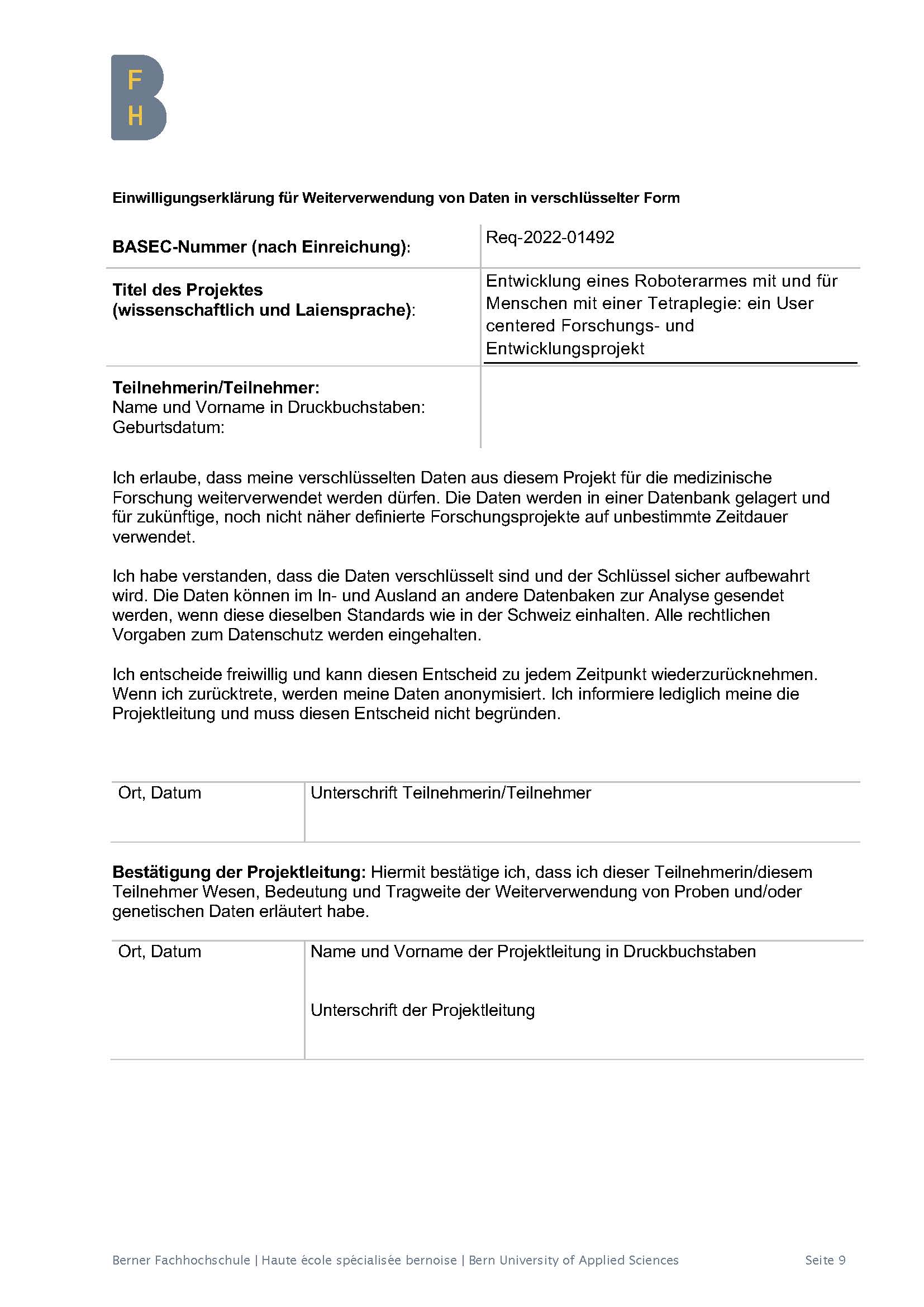

Supplement: Multimedia Appendix 12 [file rehab_v12i1e65759_app12.docx]
