# Supplementary material for: Stakeholders and Contextual Factors in the Implementation of Assistive Robotic Arms for Persons With Tetraplegia: Deductive Content Analysis of Focus Group Interviews
Source: JMIR Rehabil Assist Technol. 2025 May 16;12:e65759. doi: 10.2196/65759 (PMC12125562; doi:10.2196/65759)
Supplement: Multimedia Appendix 2 [file rehab_v12i1e65759_app2.docx]

| **Phasen** | **Minuten** | **Fragen / Inhalt** | **Bemerkungen** |
| --- | --- | --- | --- |
| **Begrüssung und Vorstellung** | 5’ |  | - Vorstellung Moderatorin inkl. Co-Moderatorin (Assistenz)  - Hinweis auf Einverständniserklärung  - Vorstellung der Ziele der Erhebung und Methodik:  Online-Regeln (Stummschaltung + Video), Fokusgruppeninterview = Leitung durch Moderatorin (Vera), nicht-direktiver Befragungsstil, sondern eine Vielfalt von Standpunkten zu dem Thema fördern.  🡪 Ich stelle es mir hier frei, falls notwendig, evtl. jemanden zu unterbrechen, damit alle zu Wort kommen ☺ Mit einigen von Ihnen bin ich per Sie / mit den anderen per Du – ich werde Sie entsprechend in diesem Sinne ansprechen, lasse es Ihnen aber offen ob Sie mich Duzen und würde hier dann aufspringen.  **Ziel**: Austausch von Perspektiven, Zusammentragen von wichtigen Aspekten für den Implementierungsprozess des assistiven Roboterarms. Dies dient dem langfristigen Ziel, dass der Roboter in einer Weise entwickelt wird, dass er schlussendlich von den Betroffenen in ihrem Alltag genutzt werden kann und im Schweizer Polit- und Versicherungssystem entsprechend finanziert/subventioniert wird.  - Focus group illustration map 🡪 wird durch Co-Moderatorin erstellt. |
| *START Video/Tonaufnahme* | | | |
| Vorstellungsrunde Stakeholder:  - Ich bitte jeden von Ihnen, sich kurz mit Namen vorzustellen und etwas zu Ihrer Funktion/Rolle in diesem Fokusgruppeninterview zu sagen | | | |
| **Einführung** | 10’ | - Vorstellen assistiver Roboterarm und Einbettung im CYBATHLON Projekt (Oktober 2024)  - Roboterarm = Prototyp, langfristig wird Effektivität im Jahr 2025 im Sinne einer klinischen Studie erhoben werden. Weiterführend noch keine Schritte als Marktprodukt durchgeführt. | - Visuelle Darstellung Roboterarm PPP  - Wäsche hängen: Pilot:In muss einen Schal über eine Wäscheleine hängen  - Spülmaschine: Pilot:In muss einen Teller aus einer geschlossenen Spülmaschine nehmen und diesen auf der Spülmaschine platzieren  - Zahnbürste: Pilot:In muss eine Zahnbürste gebrauchen  - Touchscreen: Pilot:In muss an einem Bildschirm ein vorgegebenes Produkt wählen  - Mailbox: Pilot:In muss ein Packet aus dem Briefkasten nehmen und zu einer vorgegebene Stelle transportieren  - Essen: Pilot:In muss einen Apfel aus einem Teller greifen und diesen zum Mund führen  - Auflesen: Pilot:In muss eine Petflasche vom Boden aufheben und sie aufgestellt auf dem Tisch platzieren  - Gegenstände greifen: Pilot:In muss zwei von vier unterschiedlichen Gegenständen greifen und auf eine vorgegeben Stelle platzieren  - Türe öffnen und schliessen: Pilot:In muss eine Türe öffnen, mit dem Roboterarm hindurchfahren und die Türe wieder schliessen  - Menschenmenge: Pilot:In muss mit dem Roboterarm durch einen Parcours von Möbel manövrieren und hierbei sich selbst bewegenden Robotern ausweichen |
| **Hauptteil** | 5’’ | Dies sind die Informationen, welche Sie zum assistiven Roboterarm erhalten.  - Haben Sie hierzu gerade eine konkrete Frage? |  |
| *Erster Eindruck* | 5’ | - Was halten Sie vom assistiven Roboterarm?  - Wenn Sie an Ihren Alltag denken, gibt es Aspekte, welche aktuell nicht vom Roboterarm abgedeckt sind? | - Anforderungen Alltag? |
| *Chancen* | 25’ | - Welche Chance(n) sehen Sie, wenn dieser Roboterarm in der Schweiz implementiert wird und somit für alle zugänglich gemacht wird? | - Nutzen für Betroffene/Betreuer:Innen  - Nutzen der allgemeinen Gesellschaft, wer/wie/wo? |
| *Killerkriterien/Hürden* | 25’ | - Wenn Sie sich vorstellen, dass dieser Roboterarm in der Schweiz für alle zugänglich gemacht werden soll, was muss dann gegeben sein? (Killerkriterium)  - Sehen Sie mögliche weitere Hürden, welche dem Prozess der Einführung dieses Roboters im Wege stehen? | - Effektivität  - Technische Hintergründe: wo werden sensible Daten gespeichert? Wer ist zuständig für Updates? Wer stellt Netz zur Verfügung wenn im öffentlichen Raum?  - Kosten-Nutzen Aspekt  - Ethischer Aspekt (Betreuung durch Person fällt weg) |
| *Ausblick Implementierung* | 25’ | - Welche Lösungen sehen Sie, dass die Hürden überwindet werden können?  - Wo muss/kann Zusammenarbeit stattfinden?  - Welche weiteren Rollen gibt es, die Sie als notwendig finden, um diesen Implementierungsprozess zu begleiten? |  |
| **Schluss** | 10’ | Zusammenfassung wichtigster Punkte gemäss focus group illustration map.  - Gibt es noch etwas, das ich vergessen habe und Sie gerne ergänzen würden?  - Gibt es noch etwas, dass Sie gerne sagen würden oder mir zurückmelden möchten? | - Focus group illustration map als Zusammenfassung an alle Teilnehmer:Innen per E-Mail zu einem späteren Zeitpunkt, wo Sie dem dann zustimmen können/oder ergänzen können  - Teilnehmerliste an alle Teilnehmer:Innen per E-Mail (sofern dies nicht erwünscht ist, private Email an mich) |
| *ENDE Video/Tonaufnahme* | | | |
| **Verabschiedung** | 5’ | Dank |  |
