# Supplementary material for: Stakeholders and Contextual Factors in the Implementation of Assistive Robotic Arms for Persons With Tetraplegia: Deductive Content Analysis of Focus Group Interviews
Source: JMIR Rehabil Assist Technol. 2025 May 16;12:e65759. doi: 10.2196/65759 (PMC12125562; doi:10.2196/65759)

## Multimedia Appendix 11 [Exemplary Participant Validation of Illustration Map on MS Forms (English)]


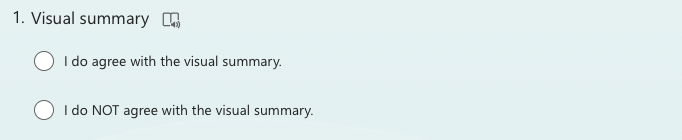


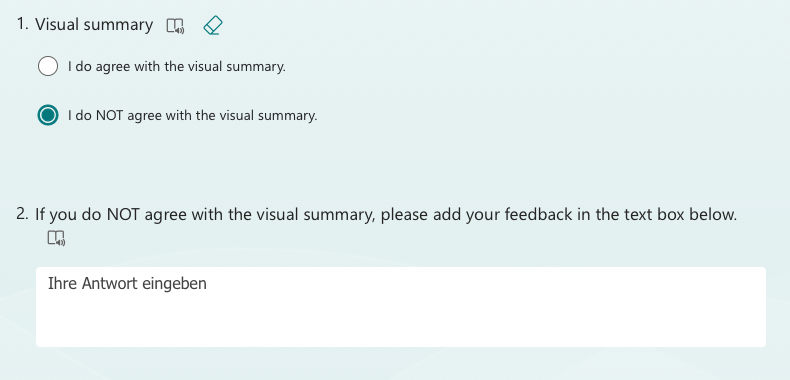

Supplement: Multimedia Appendix 11 [file rehab_v12i1e65759_app11.docx]
