# Supplementary material for: Stakeholders and Contextual Factors in the Implementation of Assistive Robotic Arms for Persons With Tetraplegia: Deductive Content Analysis of Focus Group Interviews
Source: JMIR Rehabil Assist Technol. 2025 May 16;12:e65759. doi: 10.2196/65759 (PMC12125562; doi:10.2196/65759)
Supplement: Multimedia Appendix 13 [file rehab_v12i1e65759_app13.docx]

**Table.** Barriers and facilitators identified (constructs with no barriers or facilitators were omitted).

| Constructs | | Barriers | Facilitators |
| --- | --- | --- | --- |
| **Domain I: innovation characteristics** | | | |
|  | Innovation evidence base | - No barriers | - Self-care is enabled. - Quality of life of affected persons is increased. - Resources in the care labor sector are saved. - Opportunities in the second labor market are enabled. |
|  | Innovation relative advantage | - Human interactions decrease with the use of a robot arm. - Relevant activities, such as bed or toilet transfer, are not included in the field of application of the robot arm, which limits its scope. | - Dependency on caregivers is reduced. - Dependency on social insurance pensions is reduced. |
|  | Innovation adaptability | - Use cannot be adapted to an active wheelchair. - Customization for affected persons’ needs and individual environments is a challenge. | - Customization with user interface is an opportunity. - Additional integration of an emergency system controlling physical parameters. - Combination of existing wearables with the robot arm. |
|  | Innovation trialability | - Trial testing of the robot arm needs to be possible. | - No facilitators |
|  | Innovation complexity | - Both human arm function and robotic arm function are complex. - Cognitive abilities are limiting to the use of a robot arm. - Interaction of the robot arm with the user requires safety. | - According to users, autonomy trumps speed execution. - Introduction of differing user profiles and operating modes and the automatization of the robot arm reduce complexity. - Possible shared use of the robot arm in institutions. |
|  | Innovation design | - Weight and size influence the wheelchair maneuverability. - Tipping probability of a wheelchair changes by 1-sided weight of the robot arm. - Activities that can be performed without the robotic arm are hindered - Material features of the robot arm are limiting. - Material features influence life expectancy of the robot arm. - Design is hindering or dangerous in specific environments. - Power regulation of the robot arm is limiting. - The robot arm is conspicuous in public perception. | - According to users, practicability trumps esthetics. |
|  | Innovation cost | - Current costs as a prototype are high. - Additional costs will be incurred. - Changing target group will influence the scope of the robot arm and the assumption of costs for cost bearers. | - Savings on other structural or technical aids. - Resources and costs (such as in care service) can be saved in the long term. - Current robot arm costs will decrease over time. - Work ability and integration increases. - Expansion of target group reduces costs. |
| **Domain II: the outer setting** | | | |
|  | Critical incidents | - Dependency on technology. - Warranty and availability of materials and spare parts. - Retention services, service intervals, duration of use, and guarantee pages and regulations. - Cost bearing of the robot arm. | - User involvement in the development and financing. - Public acceptance and perception of the affected person’s needs. |
|  | Local attitudes | - The robot arm is an additional aspect for stigmatization. - Lack of political and social inclusion. - Skepticism toward the robot arm. - The robot arm is not a guaranteed solution | - Technology, including robotics, is the future. |
|  | Local conditions | - The robot arm could take or cause damage in public transport. - International public transport is limited. | - Use in public transport is possible. |
|  | Partnerships and connections | - Dependency on third-party provider. | - Different stakeholders (scientific collaboration, universities, media partners, specialist retailer centers, technology partners, and physical or occupational therapists). - Political involvement. - Collaboration with specialized foundations. - Collaboration with already existing applications. |
|  | Policies and laws | - Legal authorization and certification in Switzerland. - Compliance with European MDR^a^. - Cost assumption with IV^b^ tariff allocation, and WZW-criteria^c^ need to be considered. - Registration with FSIO^d^. - Data protection for personal data. - Legal requirements for integrated camera. | - No facilitators |
|  | Financing | - The robot arm activities already provided through other AT^e^. - Cost assumption with IV: other services need to be saved. - Robot arm not assignable with current IV categories. - Already financed assistive robots by IV base on federal court judgments. - Conflicting motives between the cost bearer and the developer. - Definition of the clear area of application of the robot arm. | - Financing , in itself, is not impossible. - Cost bearers are IV, SUVA^f^, indemnity insurance, and foundations. - Step-by-step approach will simplify the assumption of costs. |
|  | External pressure | - Duplication and commercializing of prototypes. - Competition in the Swiss market. - Switzerland is a small market. | - No facilitators |
| **Domain III: the inner setting** | | | |
|  | Structural characteristics | - Individuality of the user and environmental settings require differing technical dimensions. - Adapting the setting from indoor to outdoor use seems unrealistic. | - The field of application ranges from personal household to public space. |
|  | Relational connections | - No barriers | - Psychological advantage with positive influence on the state of mind of the user and reducing tension on the personal environment. |
|  | Tension for change | - No barriers | - Already limited resources in the care labor sector are saved. |
| **Domain IV: the individuals domain** | | | |
|  | Opinion leaders | - No barriers | - Wheelchair-bound Swiss National Councilor. |
|  | Innovation deliverers | - No barriers | - Therapist, specialized retailers and distribution centers, and after-sale services. |
|  | Innovation recipients | - Unsuitable for affected persons already relying on 24-h care. | - Persons with tetraplegia. |
|  | Characteristics subdomain: need | - Safety aspects need to be considered with additional activities. - Other supplementary services are still required and should not be ruled out for the person concerned using the robotic arm. | - Independence and autonomy for affected persons. - Realization of additional activities (eg, eating independently, adjusting glasses, and shopping assistance). |

^a^MDR: Medical Device Regulation.

^b^IV: Swiss Invalidity Insurance.

^c^WZW-criteria: effectiveness, appropriateness, and economic efficiency.

^d^FSIO: Federal Social Insurance Office.

^e^AT: assistive technologies.

^e^SUVA: Swiss National Accident Insurance Fund.
